# Safety and Immunogenicity of Measles Vaccination in HIV-Infected and HIV-Exposed Uninfected Children: A Systematic Review and Meta-Analysis

**DOI:** 10.1016/j.eclinm.2018.06.002

**Published:** 2018-07-02

**Authors:** Eleonora A.M.L. Mutsaerts, Marta C. Nunes, Martijn N. van Rijswijk, Kerstin Klipstein-Grobusch, Diederick E. Grobbee, Shabir A. Madhi

**Affiliations:** a Medical Research Council, Respiratory and Meningeal Pathogens Research Unit, Faculty of Health Sciences, University of the Witwatersrand, Johannesburg, South Africa; b Department of Science and Technology/National Research Foundation, Vaccine Preventable Diseases, Faculty of Health Sciences, University of the Witwatersrand, Johannesburg, South Africa; c Division of Epidemiology and Biostatistics, School of Public Health, Faculty of Health Sciences, University of the Witwatersrand, Johannesburg, South Africa; d Julius Global Health, Julius Center for Health Sciences and Primary Care, University Medical Center Utrecht, Utrecht University, Utrecht, Netherlands; e Clinical Epidemiology, University Medical Center Utrecht, Utrecht University, Utrecht, Netherlands

**Keywords:** Measles vaccine, HIV-infection, HIV-exposure, Safety, Immunogenicity

## Abstract

**Background:**

HIV-infected and HIV-exposed uninfected (HEU) children have an increased risk of measles that may be due to altered immune responses or suboptimal timing of measles vaccination. We aimed to evaluate the safety and immunogenicity of measles vaccination in HIV-infected and HEU children.

**Methods:**

For this systematic review and meta-analysis, we searched PubMed, Embase, Cochrane Library, CINAHL, Global Health Library and IndMED on May 9, 2018. Studies were included if they reported on safety or seroresponse (either seroprotection/seropositivity/seroconversion) after measles vaccination in HIV-infected or HEU children. We calculated pooled estimates to compare immunogenicity outcomes between HIV-infected, HEU and HIV-unexposed children, using risk ratios [RRs] (with 95%CIs). PROSPERO registration number: CRD42017057411.

**Findings:**

Seventy-one studies met the inclusion criteria (15,363 children). Twenty-eight studies reported on safety; vaccine-associated adverse events and deaths were uncommon. Sixty-two studies reported on immunogenicity, 27 were included in the meta-analysis. HIV-infected children had lower seroresponse rates after primary vaccination compared with HIV-unexposed (RR 0.74; 95%CI: 0.61–0.90, *I*^*2*^ = 85.9%) and HEU children (0.78; 0.69–0.88, *I*^*2*^ = 77.1%), which was mitigated by antiretroviral therapy and time interval between vaccination and serology. HEU and HIV-unexposed children had similar seroresponses. Vaccination at 6-months resulted in similar proportions of HIV-infected children having seroresponse compared with HIV-unexposed (0.96; 0.77–1.19) and HEU children (1.00; 0.73–1.37, *I*^*2*^ = 63.7%).

**Interpretation:**

Primary measles vaccination at 6-months of age may provide protection against measles during early infancy in settings with high prevalence of maternal HIV-infection, however, further studies are needed to evaluate this strategy in HEU children and HIV-infected children receiving antiretroviral therapy.

**Funding:**

South African Research Chairs Initiative of the Department of Science and Technology and National Research Foundation in Vaccine Preventable Diseases; Medical Research Council: Respiratory and Meningeal Pathogens Research Unit.

**Research in Context**

**Evidence before this study**

Despite measles being targeted for elimination, outbreaks of measles continue to occur in low-middle income and high income countries. Contributing to this is under-immunization of children, as well as a shift in measles epidemiology towards infection of infants < 9 months of age, who are not generally targeted for measles vaccination. Young infants may be at increased risk of infection due to changes in maternal immunity, which nowadays is predominantly derived from vaccination rather than natural infection, thereby reducing transplacental transfer of protective antibodies from mother to fetus and lowering protection during early infancy. This might be further exacerbated in settings with a high prevalence of maternal HIV-infection, where there is waning of maternal immunity in HIV-infected women, that also results in lower concentrations of measles antibodies being transferred to their fetuses. Hence, HIV-exposed infants, including those who are HIV-exposed uninfected (HEU), are at increased susceptibility to measles infection during early infancy. This calls for a review of measles immunization strategy, particularly in settings with high prevalence of maternal HIV-infection, to inform future deliberations on alternate measles vaccine dosing schedule strategies.

One previous systematic review and meta-analysis on the safety and immunogenicity of measles vaccination in HIV-infected children included studies up to February 2009. Since then, antiretroviral treatment (ART) has become widely available in many countries and the number of HEU children has increased globally due to effective Prevention of Mother-to-Child Transmission programs.

We did a systematic review and meta-analysis on the safety and immunogenicity of measles vaccination in HIV-infected and HEU children. We searched seven databases (PubMed, Embase, Cochrane Library, CINAHL, Global Health Library, including African Index Medicus, Latin American and Caribbean Health Sciences, and IndMED) for articles in English, French, German, Spanish, Portuguese, or Dutch published before 9 May 2018, using the key words (“measles” and “vaccine”) and “HIV”. Reference lists of the articles that were included in full-text screening were searched manually to identify additional studies. The online database ClinicalTrials.gov was accessed for ongoing and unpublished trials. The inclusion criteria were limited to observational or interventional studies in HIV-infected or HEU children that measured safety or antibody seroresponses after measles vaccination. For inclusion in the meta-analysis a comparison group was required. Case reports were included for assessment of safety.

**Added value of this study**

The meta-analysis showed that HIV-infected children were less likely to serorespond after primary measles vaccination compared to HIV-unexposed or HEU children, while HEU and HIV-unexposed children had similar immune responses. When vaccinated at 6-months of age, similar proportions of HIV-infected and HEU children had a seroresponse compared to HIV-unexposed children. We found that vaccine-associated adverse events and deaths were uncommon.

Our study builds on the previous systematic review by incorporating additional evidence published since 2009 (nine new studies on safety and 15 new studies with comparison group on immunogenicity). To our knowledge, this is the first meta-analysis on this topic which compares measles immunogenicity outcomes considering both age at vaccination and number of doses received. We further extended previous work through detailed subgroup analyses to explore heterogeneity in seroresponse estimate and improve the robustness of the evidence by using GRADE to assess quality of evidence. HIV-infected children had a reduced immune response to primary vaccination in absence of ART, when measuring immunogenicity as seroprotection and if serology was assessed more than 3 or 6-months post-immunisation.

**Implications of all the available evidence**

In order to sufficiently protect children born to HIV-infected mothers, primary vaccination at 6-months of age is recommended. Our findings are in line with World Health Organization recommendations to administer the primary dose of measles vaccine at 6-months of age in areas with high incidence of HIV-infection and measles, followed by two routine doses according to the national immunization schedules. However, we only identified three studies evaluating measles vaccination at 6-months of age in HIV-infected and HEU children, underlining the need for further investigation before widely adopting an early vaccination strategy. Future studies should evaluate immune responses to early measles vaccination and long-term waning of immunity in HEU children and HIV-infected children treated with ART in settings with high incidence of measles and HIV.

Alt-text: Unlabelled Box

## 1 Introduction

In 2015, an estimated 1.4 million births occurred in HIV-infected women, of which more than 95% lived in low- and middle-income countries (LMICs) [1]. Increased implementation of Prevention of Mother-To-Child Transmission (PMTCT) programs has reduced vertical HIV transmission to around 1% in breastfeeding populations [2], [3] and to less than 1% in non-breastfeeding populations in LMICs [4]. As a result, a significant proportion of children born to HIV-infected mothers is HIV-exposed but uninfected (HEU). Recent studies showed that HEU children are at increased risk of morbidity and mortality compared with their HIV-unexposed peers [5], [6], [7], [8], [9], [10], [11], in particular from infectious diseases in the first 6-months of life [9], [12], [13], [14], [15], [16]. This increased susceptibility could be due to immune aberrations in HIV-exposed infants resulting from in utero exposure to HIV-virion particles or maternal antiretroviral treatment [17].

HIV-infected children have an increased risk of severe measles disease and complications compared with HIV-unexposed children [18], [19], [20]. The increased susceptibility to developing measles during early infancy in HIV-exposed infants may be explained by lower levels of maternally acquired measles antibody than HIV-unexposed [21]. Furthermore, HIV-infected, antiretroviral-naïve children have a reduced serological response to primary measles vaccination and increased waning of immunity compared with HIV-uninfected and HEU children [22], [23], [24], [25].

A previous systematic review and meta-analysis on the safety and immunogenicity of measles vaccination in HIV-infected children undertaken by Scott et al. included studies up to February 2009 [26]. Since then, the number of HEU children has increased globally and universal antiretroviral treatment for HIV-infected children is now recommended. Understanding the effects of HIV-infection and HIV-exposure on the immune response to measles vaccination is crucial for determining dosing schedules of immunisation programs, especially in LMICs with a high burden of HIV.

This systematic review evaluated the safety and immunogenicity of measles vaccine in HIV-infected and HEU children, and compared immunogenicity outcomes taking age at vaccination and number of doses received into consideration.

## 2 Methods

### 2.1 Search Strategy and Selection Criteria

This systematic review and meta-analysis adhered to the Preferred Reporting Items for Systematic Reviews and Meta-Analyses (PRISMA) guidelines [27].

We searched PubMed, Embase, Cochrane Library, Cumulative Index to Nursing and Allied Health Literature (CINAHL), Global Health Library (including African Index Medicus, Latin American and Caribbean Health Sciences), and IndMED on 9 May 2018, for articles containing (“measles” and “vaccine”) and “HIV” (Supplementary data 1). Additional studies were identified by searching reference lists of the articles included in full-text screening and ClinicalTrials.gov.

Studies were eligible for inclusion in the systematic review if they reported on immunogenicity or safety of any measles vaccination strategy in HIV-infected or HEU children aged 0–18 years. For inclusion in the immunogenicity meta-analysis, studies needed to report on primary or booster vaccination and had to include a comparator group of either HIV-uninfected children (HEU/HIV-unexposed) or HIV-infected children on a different antiretroviral therapy (ART) regimen. No restrictions regarding geographical region or year of publication were applied. Eligible study designs were interventional or observational. For assessment of safety, case reports were also included. Animal studies, systematic reviews, narrative reviews, reports of proceedings and publications not written in English, French, German, Spanish, Portuguese or Dutch were excluded.

The outcomes of interest were immunogenicity and safety. Immunogenicity: studies were included if data were reported as proportions of subjects with seroprotective (≥ 330 mIU/mL or as indicated by authors), seropositive, or seroconversion (4-fold rise in titre or change from seronegative to seropositive) measles antibody responses. A composite outcome for seroresponse was created using seroprotection rates post-vaccination, and if not available, seropositivity or seroconversion rates were considered. Safety: all reported safety outcomes post-vaccination were considered, including deaths, severe adverse events (SAEs) other than death and adverse events (AEs).

Two independent reviewers (EM, MvR) screened titles and abstracts of identified studies. Articles were retained if they met the inclusion criteria according to one or both of the reviewers. In case of duplicate publications of the same results, the most complete reference was included.

### 2.2 Data Analysis

Data were extracted from manuscripts using a standardised data extraction form (Supplementary data 2) and authors were contacted in case of missing data. Data of interest included: study design, study population, vaccine type, age at vaccination, time-period between vaccination and measurement of the serological response, number of vaccine doses administered, use of ART, outcome measures, laboratory methods used to detect measles antibodies, serological cut-off values, proportions with seroresponse, and number and type of (S)AEs.

The Cochrane Risk of Bias Tool was adapted to enable evaluation of observational studies (Supplementary data 3) [28]. For five categories, risk of bias was assessed as low (= 0), unclear (= 1), or high (= 2). Studies with a high summative risk of bias score (≥ 7) were excluded from meta-analysis.

When multiple time-points were reported for immune responses after the same vaccine dose, the time-point closest to vaccination was reported, except for two studies that had a smaller sample size at the earlier time-point [29], [30]. For the descriptive analyses, point estimates of the proportion of seroresponders for the individual studies under each group were calculated with 95% confidence intervals (CIs) assuming an exact binomial distribution.

Three different primary meta-analyses compared serological responses in HIV-infected vs. HIV-unexposed, HIV-infected vs. HEU and HEU vs. HIV-unexposed children using risk ratios (RRs) and 95%CIs stratified by vaccination dose and age at vaccination. In case of significant heterogeneity (*I*^*2*^ > 50%), a random-effects model was applied. To explore statistical variation and heterogeneity between trials, pre-specified subgroup analyses were performed based on outcome (seroprotection), serological test, use of ART, study design, age at vaccination and time interval between vaccination and measurement of the serological response. Meta-regression was used to explore between-study variance not explained by the covariates and risk of publication bias was assessed using normal and contour-enhanced funnel plots if ten or more articles were included in the meta-analysis. Small study effects were evaluated using Egger's-test for asymmetry.

We used the Grading of Recommendations Assessment, Development and Evaluation (GRADE) system for rating overall quality of evidence [31]. All analyses were performed using Stata, version 13 (StataCorpLP, Texas, USA). The study was prospectively registered in PROSPERO (CRD42017057411) [32].

### 2.3 Role of the Funding Source

The funder of the study had no role in study design, data collection, data analysis, data interpretation, or writing of the report. The corresponding author had full access to all the data in the study and all authors had final responsibility for the decision to submit for publication.

## 3 Results

**Fig. 1:**
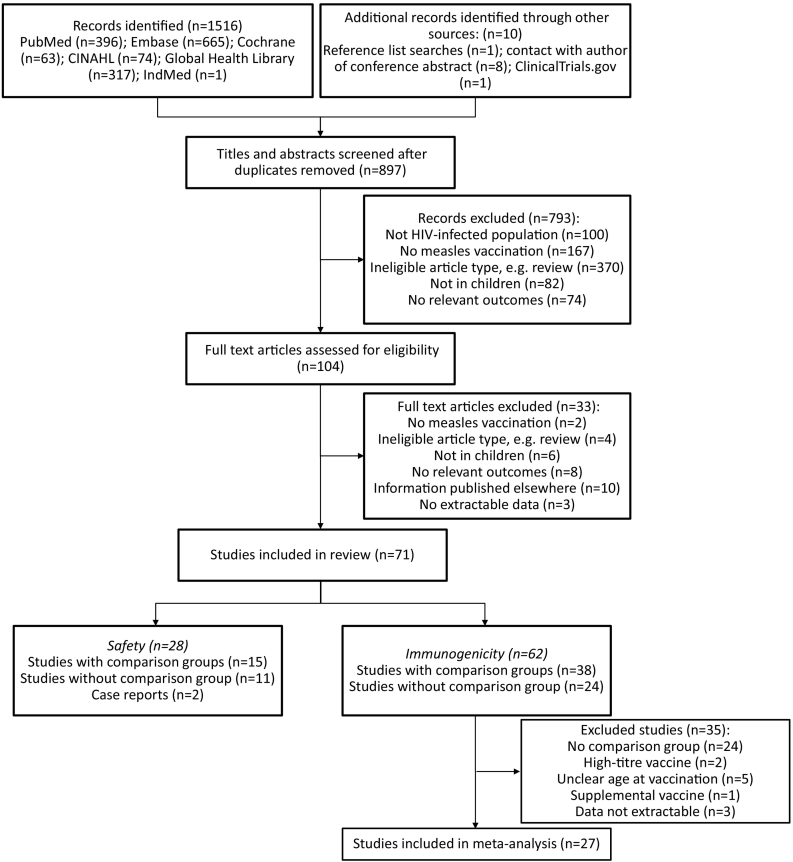
Flow chart of study selection.

We identified 897 unique articles (Fig. 1). Seventy-one studies fulfilled the eligibility criteria (Supplementary data 4). Twenty-eight studies reported on safety [24], [25], [29], [33], [34], [35], [36], [37], [38], [39], [40], [41], [42], [43], [44], [45], [46], [47], [48], [49], [50], [51], [52], [53], [54], [55], [56], [57], [58], [59], [60], [61] and 62 reported on immunogenicity [23], [24], [25], [29], [30], [33], [34], [35], [36], [37], [39], [40], [41], [43], [44], [45], [47], [48], [50], [51], [53], [55], [56], [57], [59], [60], [61], [62], [63], [64], [65], [66], [67], [68], [69], [70], [71], [72], [73], [74], [75], [76], [77], [78], [79], [80], [81], [82], [83], [84], [85], [86], [87], [88], [89], [90], [91], [92], [93], [94], [95], [96], [97], [98], [99], [100], [101], [102], [103], [104], [105], of which 27 were included in the primary meta-analyses (Table 1).

**Table 1:** Characteristics and reported proportion seroprotected/seropositive/seroconverted in the studies that assessed immunogenicity after measles vaccination included in the primary meta-analyses.

Author (year) country	Study design (start year)	Groups	Vaccine used	Age at last vaccination	Outcomes reported⁎	Interval between vaccination and serology	Number and timing of MV	Serological assay and timing of serology	Serological cut-off	Events (n)/vaccinated HIV; proportion (95%CI)	Events (n)/vaccinated HEU; proportion (95%CI)	Events (n)/vaccinated HU; proportion (95%CI)
al-Attar [62] (1995) USA	Retrospective cohort/cross-sectional (1986)	HI, HEU	Strain NR, preparation NR	1.2–2.3 yr (median 1.3 yr)	I4, I5, S0	1 mo–6.7 yr (mean 1.6 yr)	Primary vaccine? Vertically- and transfusion acquired	ELISA	Manufacturer definitions	25/40; 0.63 (0.46–0.77)	15/16; 0.94 (0.70–1.00)	
Brena [67] (1993) USA	Retrospective cohort/cross-sectional (NR)	HI, HEU	Strain NR, MMR	Median 1.3 yr (1.2–3.0 yr)	I1, I5, S0	Median 2 mo (range 1–42 mo)	Primary vaccine?	ELISA	≥ 20 EU/ml	11/20; 0.55 (0.32–0.77)	12/13; 0.92 (0.64–1.00)	
Brunell [68] (1995a) USA	Unclear (1980)	HI, HU	Strain NR, MMR/MMRV	Median 15 mo (range 8–26 mo)	I1, I5, S0	Median 7 mo (range 2–29 mo)	Primary vaccine	ELISA	OD > 42	7/9; 0.78 (0.40–0.97)		21/21; 1.00 (0.84–1.00)
Chandwani [35] (2011) USA	Randomised controlled trial (1996)	HI, HEU	Enders' attenuated Edmonston strain, MMR	Approx. 12 mo	I4, I5, S1, S2, S3	0 – approx. 2.5 yr	6 mo vaccination	PRNT, b	≥ 120 mIU/ml	7/7; 1.00 (0.59–1.00)	49/61; 0.80 (0.68–0.89)	
Chandwani [35] (2011) USA	Randomised controlled trial (1996)	HI, HEU	Enders' attenuated Edmonston strain, MMR	Approx. 12 mo	I4, I5, S1, S2, S3	0–approx. 2.5 yr	12 mo vaccination only	PRNT, b	≥ 120 mIU/ml	7/7; 1.00 (0.59–1.00)	22/22; 1.00 (0.85–1.00)	
Chandwani [35] (2011) USA	Randomised controlled trial (1996)	HI, HEU	Enders' attenuated Edmonston strain, MMR	Approx. 12 mo	I4, I5, S1, S2, S3	0–approx. 2.5 yr	6&12 mo vaccination	PRNT, b	≥ 120 mIU/ml	5/6; 0.83 (0.36–1.00)	55/56; 0.98 (0.90–1.00)	
Echeverria [39] (1996) Spain	Retrospective cohort/cross-sectional (NR)	HI, HEU	Strain NR, MMR	Approx. 12 mo	I1, S1, S2 based on adverse event statement	Approx. 1–2 yr	Primary vaccine	ELISA	> 200 mIU/ml	5/8; 0.63 (0.24–0.91)	28/30; 0.93 (0.78–0.99)	
Embree [40] (1989) Kenya	Unclear (NR)	HI, HEU	Strain NR, preparation NR	Unclear	I4, S1, S2 based on adverse event statement	Unclear	Primary vaccine?	Unclear	Protective antibody	7/8; 0.88 (0.47–1.00)	10/15; 0.67 (0.38–0.88)	
Fowlkes [43] (2011) Malawi	Prospective cohort (2000)	HI, HEU, HU	Edmonston-Zagreb, monovalent	Approx. 9 mo	I1, I6, S1, S2, S3	Approx. 3–15 mo	6 mo first dose, 9 mo serology	ELISA, b	Package insert	36/61; 0.59 (0.46–0.71)	152/223; 0.68 (0.62–0.74)	288/467; 0.62 (0.57–0.66)
Fowlkes [43] (2011) Malawi	Prospective cohort (2000)	HI, HEU, HU	Edmonston-Zagreb, monovalent	Approx. 9 mo	I1, I6, S1, S2, S3	Approx. 3–15 mo	9 mo 2nd dose, 12 mo serology	ELISA, b	Package insert	29/45; 0.64 (0.49–0.78)	189/202; 0.94 (0.89–0.97)	385/417; 0.92 (0.89–0.95)
Jain [61] (2017) India	Prospective cohort (2012)	HI, HEU, a	Edmonston-Zagreb, monovalent	Approx. 6 mo	I1, I2, S1, S2	Approx. 2–3 mo	Primary vaccine	ELISA, b	Package insert	2/6; 0.33 (0.04–0.78)	13/33; 0.39 (0.23–0.58)	
Kizito [75] (2013) Uganda	Prospective cohort (2003)	HI, HEU, HU, a?	Edmonston-Zagreb/Schwarz, monovalent	Approx. 9 mo	I6, S0	Approx. 3 mo	Primary vaccine	ELISA, b	≥ 200 mIU/ml	4/12; 0.33 (0.10–0.65)	44/62; 0.71 (0.58–0.82)	482/637; 0.76 (0.72–0.79)
Lindgren-Alves [76] (2001) Brazil	Retrospective cohort/cross-sectional (1995)	HI, HU	Strain NR, preparation NR	Unclear	I4, I5, S0	Mean 29.4 mo ± 31.9 mo	Revaccination	PRNT	> 50 mIU/ml	12/21; 0.57 (0.34–0.78)		29/29; 1.00 (0.88–1.00)
Lyamuya [78] (1999) Tanzania	Cross-sectional (1994)	HI, HU, a?	Schwarz, preparation NR	Approx. 9 mo	I5, I6, S0	Mean 26.1 mo	Primary vaccine	ELISA	≥ 200 mIU/ml	6/9; 0.67 (0.30–0.93)		617/663; 0.93 (0.91–0.95)
Molyneaux [50] (1993) UK	Retrospective cohort/cross-sectional (NR)	HI, HEU	Strain NR, monovalent or MMR	Min 1 yr	I1, S1, S2	Approx. 3–9 mo	Primary vaccine?	ELISA	Any detectable antibody	9/9; 1.00 (0.66–1.00)	61/61; 1.00 (0.94–1.00)	
Moss [51] (2007) Zambia	Prospective cohort (2000)	HI, HEU, HU	Edmonston-Zagreb, preparation NR	Approx. 9 mo	I1, I3, I5, S1, S2, S2	Approx. 1–6 mo	Primary vaccine, 6 months post-vaccination, HIV + at vaccination	PRNT, b	≥ 120 mIU/ml	44/50; 0.88 (0.76–0.95)	198/211; 0.94 (0.90–0.97)	92/98; 0.94 (0.87–0.98)
Moss [51] (2007) Zambia	Prospective cohort (2000)	HI, HEU, HU	Edmonston-Zagreb, preparation NR	Approx. 10–27 mo	I1, I3, I5, S1, S2, S2	Approx. 3–4 mo	Revaccination, 10–27 months	PRNT, b	≥ 120 mIU/ml	12/13; 0.92 (0.64–1.00)		111/115; 0.97 (0.91–0.99)
Nduati [84] (2016) Kenya	Prospective cohort (2009)	HEU, HU, a	Strain NR, preparation NR	Approx. 9 mo	I5, I6, S0	Approx. 9, 12 or 15 mo	Primary vaccine, 18 mo	ELISA	≥ 200 mIU/ml		39/47; 0.83 (0.69–0.92)	19/20; 0.95 (0.75–1.00)
Nduati [84] (2016) Kenya	Prospective cohort (2009)	HEU, HU, a	Strain NR, preparation NR	NR	I5, I6, S0	Approx. 9, 12 or 15 mo	Primary vaccine?, > 18 mo	ELISA	≥ 200 mIU/ml		8/8; 1.00 (0.63–1.00)	26/28; 0.93 (0.76–0.99)
Oxtoby [55] (1989) Zaire	Prospective cohort (NR)	HI, HEU, HU	Strain NR, preparation NR	Approx. 9 mo	I2, S1, S2, S3	Approx. 12 mo	Primary vaccine	Unclear	Seronegative to Seropositive	24/37; 0.65 (0.47–0.80)	140/157; 0.89 (0.83–0.94)	199/224; 0.89 (0.84–0.93)
Pensieroso [89] (2009) Italy	Cross-sectional (NR)	HI, HU, a	Schwarz, MMR	Approx. 13–15 mo	I2, I6, S0	Mean 4.7 yr	Primary vaccine	ELISA	≥ 200 mIU/ml	33/70; 0.47 (0.35–0.59)		50/50; 1.00 (0.93–1.00)
Rainwater-Lovett [92] (2013) Zambia	Prospective cohort (2008)	HI, HU (presumed), a	Strain NR, preparation NR	Median 10 mo	I1, I2, S0	Median 11 mo	Primary vaccine	ELISA	> 120 mIU/ml	46/116; 0.40 (0.31–0.49)		9/12; 0.75 (0.43–0.95)
Rainwater-Lovett [92] (2013) Zambia	Prospective cohort (2008)	HI, HU (presumed), a	Strain NR, preparation NR	Median 10 mo	I1, I2, S0	Median 11.0 mo	Revaccination	ELISA	> 120 mIU/ml	18/19; 0.95 (0.74–1.00)		13/13; 1.00 (0.75–1.00)
Reikie [93] (2013) South Africa	Prospective cohort (2009)	HEU, HU	Strain NR, preparation NR	Approx. 18 mo	I5, I6, S0	Approx. 3, 9, 13 mo	Primary vaccine, 12 mo serology	ELISA, b	≥ 330 mIU/ml		22/27; 0.81 (0.62–0.94)	20/28; 0.71 (0.51–0.87)
Reikie [93] (2013) South Africa	Prospective cohort (2009)	HEU, HU	Strain NR, preparation NR	Approx. 18 mo	I5, I6, S0	Approx. 3, 9, 13 mo	Two doses, 24 mo serology	ELISA	≥ 330 mIU/ml		19/27; 0.70 (0.50–0.86)	13/27; 0.48 (0.29–0.68)
Rudy [59] (1994a) USA	Unclear (1990)	HI, HEU	Strain NR, monovalent	6–11 mo	I4, S1, S2	Approx. 1–3 mo	Primary vaccine, monovalent < 12 mo	ELISA, b	Unclear	9/13; 0.69 (0.39–0.91)	17/22; 0.77 (0.55–0.92)	
Rudy [59] (1994b) USA	Unclear (1990)	HI, HEU	Strain NR, MMR	12–15 mo	I4, S1, S2	Approx. 1–3 mo	Primary vaccine MMR ≥ 12 mo	ELISA, b	Unclear	6/12; 0.50 (0.21–0.79)	13/14; 0.93 (0.66–1.00)	
Siberry [96] (2015) USA	Prospective cohort (2007)	HI, HEU, a	Edmonston-Zagreb, MMR	Median 4.32 yr (IQR 4.04–5.03 yr)	I6, S0	Median 9.8 yr (IQR 6.9–12.1 yr)	Revaccination (for 2% primary vaccine)	PRNT	≥ 120 mIU/ml	244/428; 0.57 (0.52–0.62)	219/221; 0.99 (0.97–1.00)	
Simani [97] (2013) South Africa	Prospective cohort (archived serum samples) (2005)	HI, HEU, HU	Schwarz, monovalent	Mean 67.8 wks ± 4.4	I1, I5, I6, S0	28 wks post MV1	Primary vaccine, 28 wks post-primary, HIV groups combined	ELISA	≥ 330 mIU/ml	225/253; 0.89 (0.84–0.93)	110/116; 0.95 (0.89–0.98)	102/112; 0.91 (0.84–0.96)
Simani [97] (2013) South Africa	Prospective cohort (archived serum samples) (2005)	HI, HEU, HU, a	Schwarz, monovalent	Mean 67.8 wks ± 4.4	I1, I5, I6, S0	28 wks post MV1, 2 and 41 wks post MV2	Two doses, 2 wks post-booster, def-ART	ELISA, b	≥ 330 mIU/ml	235/248; 0.95 (0.91–0.97)	104/114; 0.91 (0.84–0.96)	111/115; 0.97 (0.91–0.99)
Succi [105] (2018) Latin America and the Caribbean	Prospective cohort	HI, HEU, a	Strain NR, preparation NR	Approx. 1 yr	I1, I5, S0	Approx. 2.8 yrs	Primary vaccine	ELISA	≥ 120 mIU/ml	77/96; 0.80 (0.71–0.88)	51/51; 1.00 (0.93–1.00)	
Sudfeld [100] (2013) Tanzania	Prospective cohort (2005)	HI, HEU, a?	Edmonston-Zagreb, preparation NR	Approx. 9 mo (9–12 mo)	I1, I5, S0	Approx. 3–10 mo	Primary vaccine	ELISA, b	≥ 200 mIU/ml	16/35; 0.46 (0.29–0.63)	138/201; 0.69 (0.62–0.75)	
Tejiokem [103] (2007) Cameroon, Central African Republic	Cross-sectional (2004)	HI, HEU, a	Strain NR, preparation NR	9 mo–1.3 yr	I1, I5, S0	Median 12.8 mo (90% range; 3.3–26.1 months)	Primary vaccine, commercial ELISA kit	ELISA, b	≥ 335 mIU/ml	7/46; 0.15 (0.06–0.29)	45/72; 0.63 (0.50–0.74)	
Tejiokem [103] (2007) Cameroon, Central African Republic	Cross-sectional (2004)	HI, HEU, a	Strain NR, preparation NR	9 mo-1.3 yr	I1, I5, S0	Median 12.8 mo (90% range; 3.3–26.1 months)	Revaccination, commercial ELISA kit	ELISA, b	≥ 335 mIU/ml	1/4; 0.25 (0.01–0.81)	3/5; 0.60 (0.15–0.95)	
Thaithumyanon [25] (2000) Thailand	Prospective cohort (NR)	HI, HEU	Schwarz, monovalent	Approx. 9 mo	I2, I5, S1, S2, S3	Approx. 12 wks	Primary vaccine	ELISA, b	> 150 mIU/ml	8/14; 0.57 (0.29–0.82)	14/14; 1.00 (0.77–1.00)	
Waibale [104] (1999) Uganda	Retrospective cohort/cross-sectional (1995)	HI, HEU	Strain NR, monovalent	Median 9.4 mo (5.2–25.8 mo)	I1, I5, S0	Median 14 mo (2.7–30.8 mo)	Primary vaccine (99%)	ELISA	≥ 15 EU/ml	24/50; 0.48 (0.34–0.63)	122/193; 0.63 (0.56–0.70)	
Walter [30] (1994) USA	Retrospective cohort/cross-sectional (1992)	HI, HEU	Strain NR, MMR	Mean 20.4 month (± 10.2 mo)	I4, I5, S0	Mean 13.3 mo	Unclear, mean 13.3 m post-vaccination	ELISA	≥ 0.065 OD	14/20; 0.70 (0.46–0.88)	11/11; 1.00 (0.72–1.00)	

HEU, HIV-exposed uninfected; HI, HIV-infected; HU, HIV-unexposed; ELISA, enzyme-linked immunosorbent assay; EU/ml, ELISA units per milliliter; mIU/ml, milli international units per milliliter; mo, months of age; MV, measles vaccination; MMR, measles, mumps, rubella vaccine; MMRV, measles, mumps, rubella, varicella vaccine; NA, not applicable; NR, not reported; OD, optical density; PRNT, plaque reduction neutralization test; sMV, supplemental measles vaccination; yr, years of age.

a: studies where children received antiretroviral therapy.

a?: studies where it is not clear if children received antiretroviral therapy.

b: studies where blood was drawn for measles serology less than six months after vaccination.

⁎ I Immunogenicity outcomes: I0, immunogenicity not reported; I1, Seropositivity after vaccination reported; I2, seroconversion (seronegative before vaccination, Seropositive after vaccination) reported; I3, seroconversion (4-fold rise in titre) reported; I4, measure, which might be either Seropositivity, seroconversion or seroprotection after vaccination, is reported; I5, summary immunological measure (e.g. geometric mean titre) reported; I6, seroprotection after vaccination reported; S Safety outcomes: S0, no adverse event information reported; S1, explicit reporting on adverse events; S2, explicit reporting on serious adverse events; S3, reporting on deaths.

Included study designs were randomised controlled trial (RCT) (n = 1) [35], [36], cohort (n = 35), cross-sectional (n = 30), case reports (n = 2) [46], [58], retrospective audits (n = 1) [72]; two studies had an unclear study design [40], [68]. Studies were published from 1987 through 2018 and were conducted in Africa (n = 28), the United States (n = 16), Europe (n = 17), South America (n = 5) and Asia (n = 5).

Taking all studies together, 15,363 children vaccinated against measles were evaluated, of which 4867 were HIV-infected, 2733 were HEU, and 7763 were HIV-unexposed.

Thirty-five studies with comparison groups reported post-vaccination seroresponses in HIV-infected children, of which twelve administered ART (Supplementary data 5.1). HIV-infected children showed similar seroresponse rates after primary vaccination at 6-months (pooled estimate 71%; 95%CI 55–88; n = 5) compared with later time points: 9-months (60%; 95%CI 43–77; n = 12), 12-months (84%; 95%CI 48–120; n = 2) and > 12-months of age (64%; 95%CI 51–76; n = 7). The pooled point estimate of HIV-infected children with seroresponse after booster vaccination was similar when administered at ≤ 24 months (77%; 95%CI 58–96; n = 5) or > 24 months (61%; 95%CI 39–83; n = 4). Two studies assessed the effect of different ART-regimens on the response to primary vaccination [97], [105] and four studies to booster vaccination [66], [70], [89], [97]. Children receiving ART or early-ART within the first year of life showed improved seroresponses to booster vaccination compared with those who received late-ART or did not receive ART [66], [70], [89].

HEU children receiving primary vaccination at 12-months (pooled estimate 98%; 95%CI 91–104; n = 2) or > 12-months of age (99%; 95%CI 96–102; n = 5) tended to have better seroresponse compared with HEU children vaccinated at 6 (70%; 95%CI 58–83; n = 5) or 9-months (84%; 95%CI 76–91; n = 13) of age (Supplementary data 5.2).

Similar to HEU children, a trend towards improved seroresponse was observed in HIV-unexposed children receiving primary vaccination at > 12-months (pooled estimate 100%; 95%CI 97–103; n = 2) compared with 6-months (66%; 95%CI 50–82; n = 3) or 9-months of age (88%; 95%CI 82–94; n = 9) (Supplementary data 5.3).

**Fig. 2:**
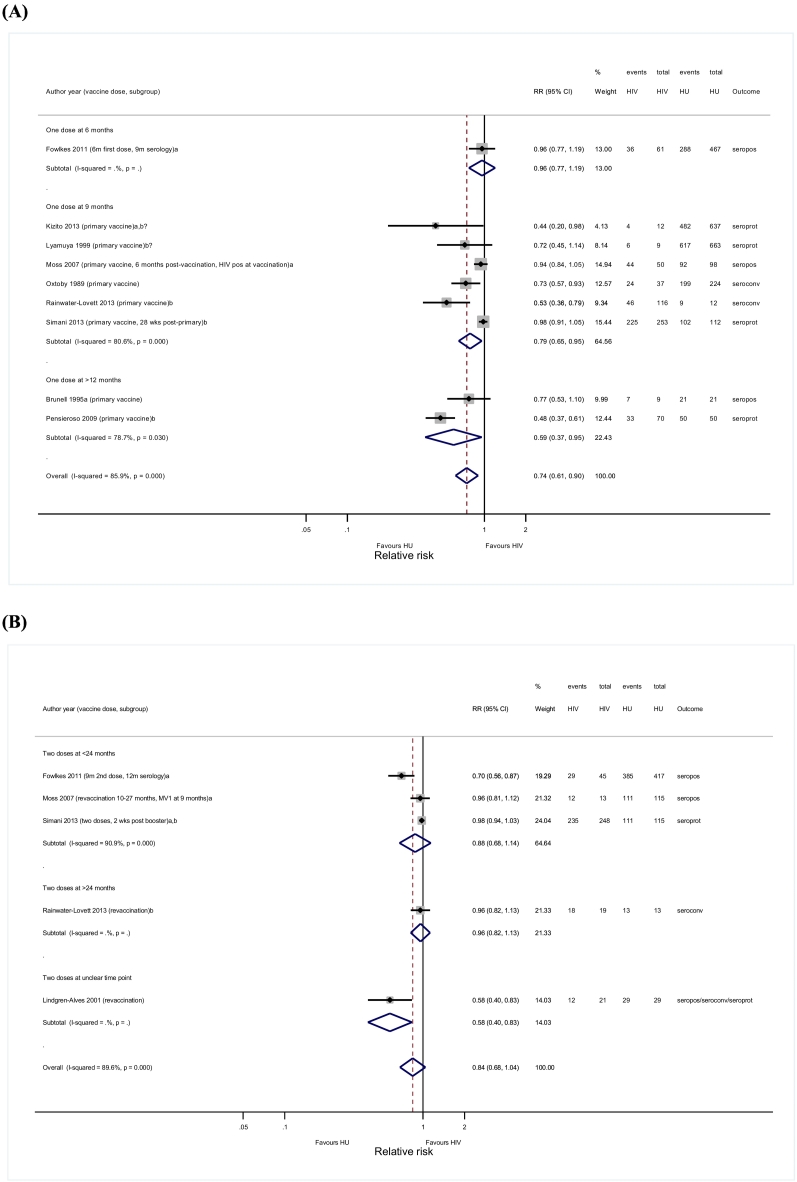
Forest plots for seroresponses comparing HIV-infected and HIV-unexposed children. (A) One dose of measles vaccine; (B) Two or more doses of measles vaccine. ART, antiretroviral therapy; HU, HIV-unexposed; RR, Risk Ratio; seroconv, seroconversion; seropos, seropositivity; seropos/seroconv/seroprot, might either be seropositivity, seroconversion or seroprotection; seroprot, seroprotection; a: studies where blood was drawn for measles serology within six months after vaccination; b: studies where children received antiretroviral therapy; b?: studies where it is not clear if children received antiretroviral therapy.

Nine publications were included in the primary meta-analysis comparing immune responses after primary vaccination in HIV-infected and HIV-unexposed children [43], [51], [55], [68], [75], [78], [89], [92], [97]. Relative risks for all studies were < 1, although only significant in four studies [55], [75], [89], [92]. ART was administered in two of four studies with a significant RR [89], [92], compared with one of five studies that did not find a significant difference [97]. The pooled RR resulting from the random-effects model was 0.74 (95%CI 0.61–0.90; *I*^*2*^ = 85.9%) (Fig. 2A). Seroresponses after primary vaccination at 9-months (RR = 0.79; 95%CI 0.65–0.95) and > 12-months of age (RR = 0.59; 95%CI 0.37–0.95) were significantly lower in HIV-infected compared with HIV-unexposed children, but not when vaccinated at 6-months (RR = 0.96; 95%CI 0.77–1.19; n = 1). Limiting analysis to studies that reported seroprotection (RR = 0.64; 95%CI 0.36–1.14; n = 4), administered ART (RR = 0.63; 95%CI 0.34–1.19; n = 3), or measured serology within 3 (RR = 0.71; 95%CI 0.33–1.55; n = 2) or 6-months post-vaccination (RR = 0.90; 95%CI 0.73–1.11; n = 3), resulted in non-significant combined RRs (Supplementary data 6 and 8.1).

Meta-analysis in five studies comparing post-booster responses in HIV-infected and HIV-unexposed children found a pooled non-significant RR (0.84, 95%CI 0.68–1.04; *I*^*2*^ = 89.6%) (Fig. 2B), irrespective of subgroup analyses (Supplementary data 7 and 8.2) [43], [51], [76], [92], [97].

**Fig. 3:**
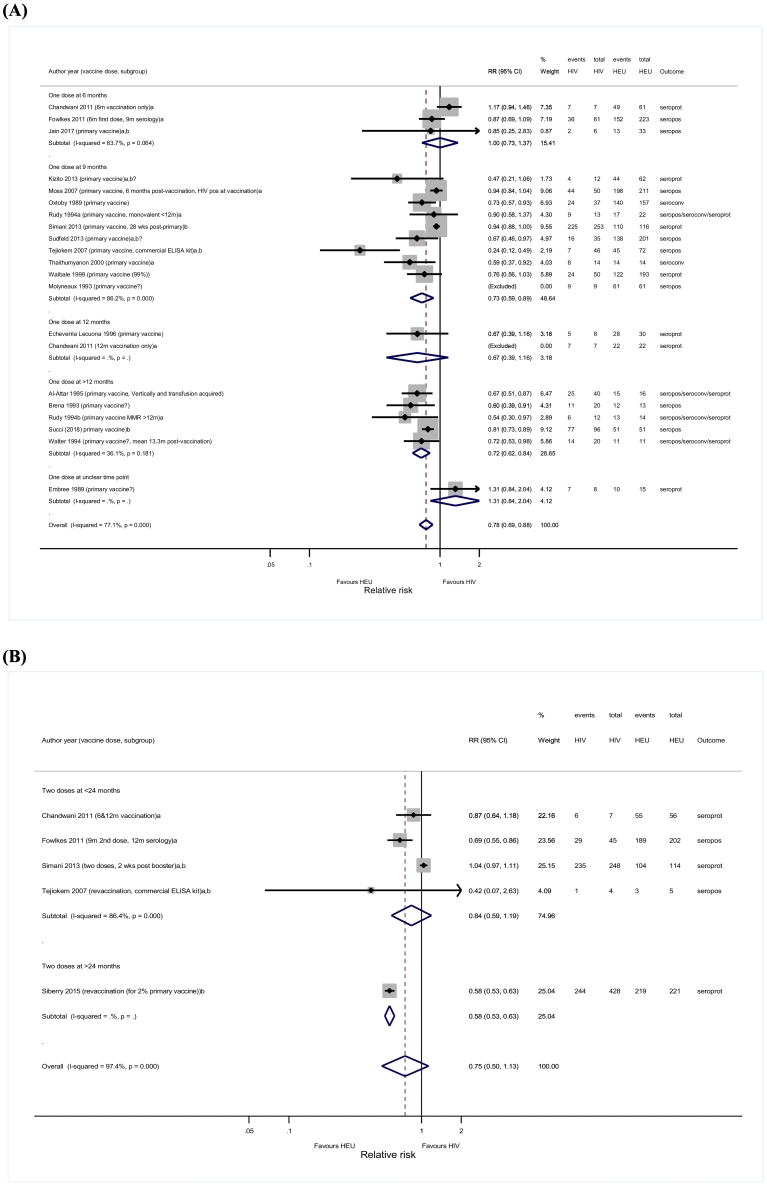
Forest plots for seroresponses comparing HIV-infected and HIV-exposed uninfected children. (A) One dose of measles vaccine; (B) Two or more doses of measles vaccine. ELISA, enzyme-linked immunosorbent assay; HEU, HIV-exposed uninfected; MMR; Measles Mumps Rubella; RR, Risk Ratio; seroconv, seroconversion; seropos, seropositivity; seropos/seroconv/seroprot, might either be seropositivity, seroconversion or seroprotection; seroprot, seroprotection; a: studies where blood was drawn for measles serology within six months after vaccination; b: studies where children received antiretroviral therapy; b?: studies where it is not clear if children received antiretroviral therapy.

Twenty-one studies compared immunogenicity after primary measles vaccination between HIV-infected and HEU children. Nine studies reported significant RR estimates < 1 [25], [30], [55], [59], [62], [67], [100], [103], [105], two included HIV-infected children on ART [103], [105]. The pooled RR comparing HIV-infected and HEU children after primary measles vaccination was 0.78 (95%CI 0.69–0.88; *I*^*2*^ = 77.1%) (Fig. 3A). The proportion of HIV-infected children with seroresponse after primary vaccination was lower compared with HEU when vaccinated at either 9-months (RR = 0.73; 95%CI 0.59–0.89; n = 10) or > 12-months of age (RR = 0.72; 95%CI 0.62–0.84; n = 5), but not at 6-months (RR = 1.00; 95%CI 0.73–1.37; n = 3) of age. The combined RRs followed the same trend when limiting analysis to studies that administered ART (RR = 0.74; 95%CI 0.54–1.00; n = 4), analysed serology within 3-months post-vaccination (RR = 0.79; 95%CI 0.60–1.04; n = 8), or reported seroprotection (RR = 0.92; 95%CI 0.74–1.15; n = 7), although non-significant (Supplementary data 6 and 8.3). Random effects meta-regression identified significant subgroup differences for studies with a different serological outcome measure (1.17; 95%CI 1.05–1.31), which could explain about 40% of between-study variance.

HIV-infected and HEU children showed similar immune responses after booster measles vaccination (RR = 0.75; 95%CI 0.50–1.13; n = 5) (Fig. 3B) [35], [43], [96], [97], [103]. When stratified by age at vaccination, HIV-infected children were less likely to show a seroresponse when vaccinated at > 24 months (RR = 0.58; 95%CI 0.53–0.63; n = 1), but not at ≤ 24 months of age (RR = 0.84; 95%CI 0.59–1.19; n = 4). Pooled RRs in subgroup analyses yielded similar results (Supplementary data 7 and 8.4).

**Fig. 4:**
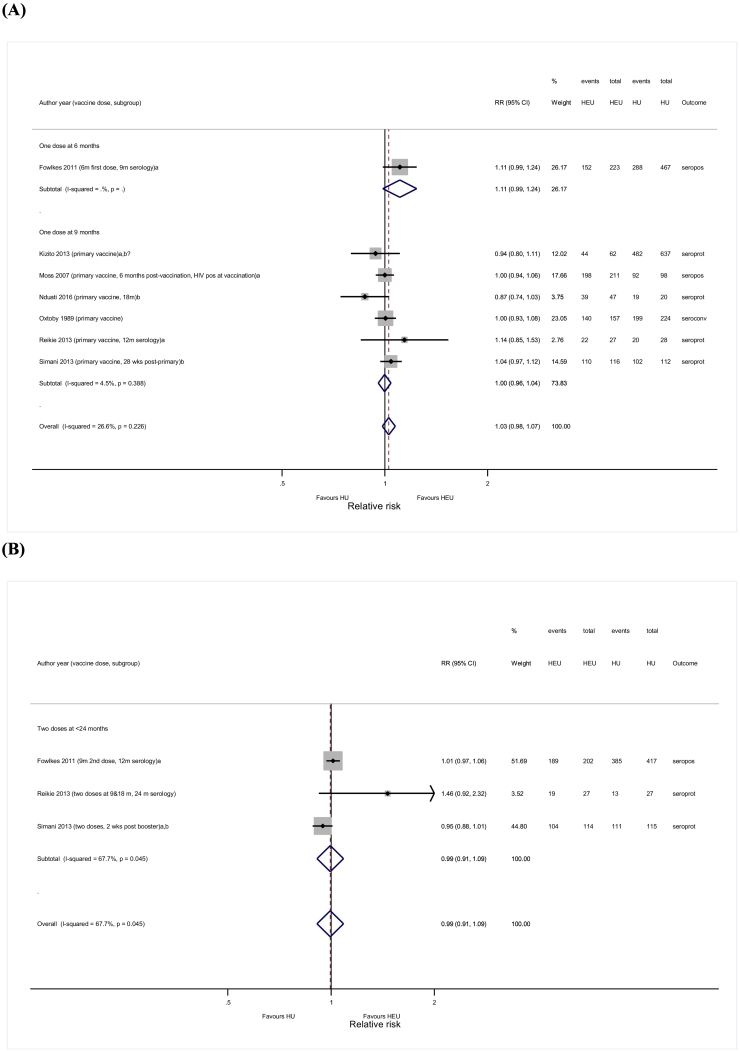
Forest plots for seroresponses comparing HIV-exposed uninfected and HIV-unexposed children. (A) One dose of measles vaccine; (B) Two or more doses of measles vaccine. HEU, HIV-exposed uninfected; HU, HIV-unexposed; RR, Risk Ratio; seroconv, seroconversion; seropos, seropositivity; seroprot, seroprotection; a: studies where blood was drawn for measles serology within six months after vaccination; b: studies where children received antiretroviral therapy; b?: studies where it is not clear if children received antiretroviral therapy.

None of the seven studies reporting on immunogenicity outcomes after primary vaccination in HEU and HIV-unexposed children [43], [51], [55], [75], [84], [93], [97] found significant differences between the two groups. The pooled RR from a fixed-effects model showed similar seroresponses between HEU and HIV-unexposed children (RR = 1.03; 95%CI 0.98–1.07; *I*^*2*^ = 26.6%), irrespective of age or other covariates (Fig. 4A, Supplementary data 6 and 8.5).

The meta-analysis comparing HEU to HIV-unexposed children after booster vaccination showed a similar likelihood of seroresponding among the two groups (R = 0.99; 95%CI 0.91–1.09; *I*^*2*^ = 67.7%) (Fig. 4B) [43], [93], [97].

Twenty-eight studies reported on safety (Table 2). In total, 102 HIV-infected and 21 HIV-uninfected children died after immunisation. For two deaths in HIV-infected children, the relation between vaccine administration and death could not be definitely ascertained, of which one occurred within a month post-vaccination [49], [51]. The median time between vaccine administration and end of study during which monitoring of deaths was performed was 38 weeks (range 4–144 weeks).

**Table 2:** Adverse events, serious adverse events and deaths in studies reporting on safety.

Study	AEs in HIV-infected/total HIV-infected	AEs in HEU/total HEU	AEs in HIV-unexposed/total HIV-unexposed	SAEs (other than death) in HIV-infected/total HIV-infected	SAEs (other than death) in HEU/total HEU	SAEs (other than death) in HIV-unexposed/total HIV-unexposed	Vaccine-related SAEs (other than death) in HIV-infected	Time observed for SAEs other than death	Post-vaccination deaths in HIV-infected/total post-vaccination deaths in all groups	Vaccine related potentially life-threatening events or deaths	Time observed for deaths
Abzug [33] 2012	NR	–	–	4/193	–	–	NR	28 days	NR	NR	–
Aurpibul [34] 2007	23/51	–	–	0/51	–	–	NA	28 days	NR	–	–
Chandwani [35] 2011a (& Chandwani [36] 1998)	4/8	9/27	–	0/8	0/27	–	0	14 days	0/0	NA	NR
Chandwani [35] 2011b (& Chandwani [36] 1998)	2/7	17/61	–	0/7	0/61	–	0	14 days	0/0	NA	NR
Cutts [37] 1993	29/49^a^	18/376^b^		9/49	4/376		0	5–15 days	9/13	0	Median 1.7 years
Dunn [38] 1998	NR	NR	–	0/56	1/616	–	0	NR	NR	–	–
Echeverria Lecuona [39] 1996	10/14	NR	–	0/14	NR	–	NR	NR	0/NA	NA	NR
Embree [40] 1989	NR	NR	–	0/unclear	0/unclear	–	NA	NR	NR	–	–
Farquhar [41] 2009	NR	–	–	NR/18	–	–	–	NR	0/NA	NA	NR
Fernandez-Ibieta [42] 2007	NR	–	–	NR/55	–	–	–	NR	0/NA	NA	NR
Fowlkes [43] 2011 (& Helfand [24] 2008)a	31/83^c^	84/246^c^	186/512^c^	NER	NER	NER	0	28 days	34/NER	0	16.5 months
Fowlkes [43] 2011 (& Helfand [24] 2008)b	25/59^d^	80/222^d^	152/453^d^								
Fowlkes [29] 2016	NR	NR	NR	0/22	NR	0/865	NA	21 days	NER	0	36 months
Frenkel [44] 1994 (Frenkel [45] 1992)	NR	–	–	0/10	–	–	NA	NR	NR	–	–
Goon [46] 2001	NR	–	–	1/1	–	–	NR	10 days	0/NA	NA	1 year
Jain [61] 2017	2/7	5/39	–	NR	NR	–	0	28 days	NER	0	1 month
Lepage [46] 1992	20/36	71/121	68/166	0/36	1/121	0/166	0	8–14 days	15/17	0	18 months
Marczynska [48] 2001 (substudy)	NR	–	–	0/9	–	–	NA	28 days	0/0	NA	3 months
McLaughlin [49] 1988	NR	–	–	1/70	–	–	Potentially 1, but relation to vaccination not verifiable	NR	Unclear, 41 of 221 HIV-infected patients (19%) died (vaccinated and unvaccinated)/NA	Potentially 1, but relation to vaccination not verifiable	NR
Molyneaux [50] 1993	NR	NR	–	1/9^e^	0/61	–	NA	NR	NR	–	–
Moss [51] 2007	41% of 66 with fever, 70% of 66 with cough	NR	41% of 375 with fever, 57% of 375 with cough	1/66	NR	2/375	NR	28 days	28/38	1 died with measles, but not known to be related to vaccination	27 months
Ndikuyeze [52] 1987	NR	–	–	0/3	–	–	NA	NR	NR	NA	–
Oldakowska [53] 2001	0/13	–	–	0/13	–	–	NA	28 days	NR	–	–
Oshitani [54] 1996	NR	–	NR	11/37	–	5/111	NR	NR	11/16	NR	NR
Oxtoby [54] 1989	NR	NR	NR	4/37^f^		11/381^f^	NER	NR	NER	–	NR
Palumbo [56] 1992 (& Hoyt [57] 1992)	0/92^g^	–	–	4/94	–	–	NR	NR	2/NA	0	NR
Ramon-Garcia [58] 1995	NR	–	–	2/2	–	–	NR	NR	2/NA	NR	NR
Rudy [59] 1994a&b	0/13 and 0/12	0/22 and 0/14	–	0/13 and 0/12	0/22 and 0/14	–	NA	NR	NR	–	–
Seth [60] 2016	0/66	–	–	0/66	–	–	NA	28 days	NR	–	–
Thaithumyanon [25] 2000	NR	NR	–	NR	NR	–	–	short term	1/NER	0	12 weeks

Studies were excluded from the safety table if they did not report on serious adverse events or deaths.

AE, adverse event; HEU, HIV-exposed uninfected; HI, HIV-infected; HU, HIV-unexposed; HU, HIV-unexposed; NA, not applicable; NER, not explicitly reported; NR, not reported; SAE, serious adverse event.

a Incidence of symptoms with onset within 5–15 days after vaccination among HIV-infected infants: diarrhoea (n = 22), cough (n = 14), rhinorrhoea (n = 12), fever (n = 29), morbilliform rash (n = 2), unscheduled consultation (n = 6); highest number (n = 29) used for calculations.

b Incidence of symptoms with onset within 5–15 days after vaccination among non-HIV-infected infants: diarrhoea (n = 14), cough (n = 15), rhinorrhoea (n = 13), fever (n = 18), conjunctivitis (n = 3), unscheduled consultation (n = 7); highest number (n = 18) used for calculations.

c Parental reports of any symptoms during the first 21 days after measles vaccination at 6 months of age.

d Parental reports of any symptoms during the first 21 days after measles vaccination at 9 months of age.

e HIV-infected child who required hospital admission for severe measles, but unclear whether this was before or after vaccination.

f Only cases of clinical measles explicitly reported during follow-up at a mean of 9 months after vaccination.

g Unclear number of HIV-infected children vaccinated in case finding; number reported during outbreak.

Twenty-three studies provided information on post-vaccination SAEs other than death in HIV-infected children (period of observation ranged 1–4 weeks post-vaccination). SAEs other than death were reported in 29 of 884 HIV-infected children (3.3%), 2 of 1337 HEU (0.1%), and 18 of 1898 HIV-unexposed children (0.9%). None of the verifiable SAEs were vaccine-related. One study reported a possible, but unverifiable vaccine-related SAE [49]. HIV-uninfected children were more likely to experience AEs (41%) compared with HIV-infected (33%) or HEU (25%) children (p < 0.001) (Supplementary data 9).

**Fig. 5:**
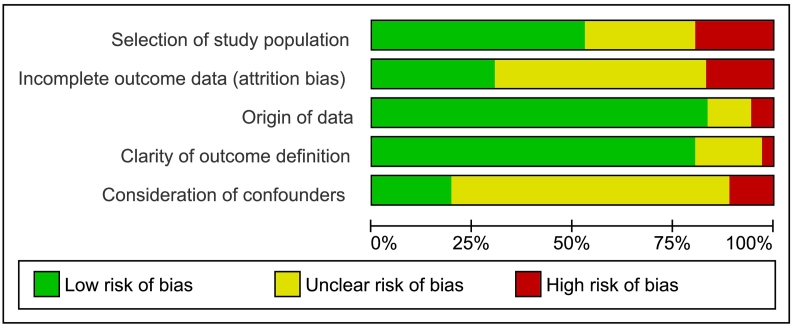
Summary of risk of bias evaluation using adapted Cochrane framework.

Of the 71 studies, 59 (83%) had unclear or high-risk of confounding bias and 55 (77%) had unclear or high-risk of attrition bias due to incomplete outcome data. The origin of data and the clarity of outcome definition had low-risk of bias in 60 (85%) and 54 (76%) studies, respectively (Fig. 5, Supplementary data 10). No studies had a high summative risk of bias score (≥ 7).The GRADE quality of evidence was low or very low, except for the included RCT (Supplementary data 11–14).

The funnel plot for comparisons containing ten or more studies (HIV-infected vs. HEU children after primary vaccination) had an asymmetrical appearance (Supplementary data 15a). The contour-enhanced funnel plot showed that studies were missing in regions of both low and high statistical significance (Supplementary data 15b), suggesting that the asymmetry cannot be explained by publication bias. Smaller studies were likely to have contributed to funnel plot asymmetry (Egger's test p = 0.009).

## 4 Discussion

This review assessed the safety and immunogenicity of measles vaccination in 4867 HIV-infected, 2733 HEU and 7763 HIV-unexposed children. HIV-infected children had 26% (95%CI 10%–39%) lower seroresponse rate to primary measles vaccination compared with HIV-unexposed children, and 22% (95%CI 12%–31%) lower rate compared with HEU children. Differences between groups were no longer present after booster vaccination [25], [63], [106]. This might be due to selection of HIV-infected children that survived to an older age, who were likely to be slow progressors and maintained their immunological status, or received ART. No association between death and measles vaccination was found in HIV-infected children. None of the verifiable SAEs were vaccine-related.

Primary measles vaccination with standard titre measles vaccine at 6-months of age resulted in similar seroresponse rates between groups of HIV-infected [43], HEU [35], [43], and HIV-unexposed children. This finding is supported by studies using high-titre primary measles vaccination at 6-months [37], [47].

Pooled RRs showed no difference between HIV-infected and HIV-unexposed or HEU children after primary vaccination when limiting the meta-analysis to studies that administered ART, reported on seroprotection, or measured serology within 3 or 6-months post-vaccination. Thus, reduced seroresponse to primary vaccination may particularly be evident in HIV-infected children when using a less stringent serological cut-off (seroconversion or seropositivity instead of seroprotection), in the absence of ART, or after a longer time-period between vaccination and serology.

Studies with different timing for ART initiation showed improved immune responses to booster vaccination in HIV-infected children after ART initiation [34], [41], [66], [79] or when started on early-ART [70], [89], [97], while late- or non-treated groups had reduced protective responses after revaccination.

HIV-exposed children showed a non-significant trend towards improved serological response when vaccinated at 6-months of age compared with HIV-unexposed children. This could be explained by reduced transplacental transfer of antibodies from HIV-infected women, resulting in lower levels of maternal antibodies in the infant and less interference with the B-cell response to vaccination [21]. Maternal PMTCT regimens and breastfeeding recommendations for HIV-infected mothers varied substantially between 1987 and 2018, and may have contributed to differences between HEU and other groups. Fetal ART exposure has been associated with less hypergammaglobulinemia in HEU children [107] and higher transfer of transplacental pathogen-specific antibodies was reported in women on triple ART compared with women on short course zidovudine [108]. In this meta-analysis, only two studies reported on maternal ART [61], [100] and one on breastfeeding [100]; no association with measles seroresponse was found.

HIV-infected children experienced slightly more SAEs other than death in the first 4-weeks post-vaccination compared with HEU or HIV-unexposed children. However, due to absence of direct comparisons between vaccinated and unvaccinated HIV-infected children and poor quality of reporting, limited conclusions can be drawn from this analysis. HIV-infected children may experience more SAEs due to their underlying illness, unrelated to vaccine administration.

A previous systematic review and meta-analysis of 39 studies analysing safety and immunogenicity of measles vaccination in HIV-infected children searched literature up to February 2009 [26]. The analysis was not stratified according to primary or booster vaccination. We included nine new studies on safety and 15 new studies on immunogenicity. In line with the previous review, we found a trend towards improved serological responses with increasing age at vaccination in HEU and HIV-unexposed children in the descriptive analysis.

Strengths of this review and meta-analysis are the comprehensive search in seven databases and the large number of studies identified. Also, this is the first meta-analysis on this topic to separately analyse primary and booster dose by age at vaccination.

Our results need to be interpreted in the context of the risk of bias evaluation and low to very low quality of evidence. All studies included in this review were of observational nature, except for one RCT [35], [36]. Observational studies may be subject to selection and confounding bias. The majority of studies did not account for age, time since vaccination and CD4 + T-cell count, hence unadjusted outcome measures were used in the analysis. A large number of studies were cross-sectional, and single time-point data were used for assessment of immune responses, increasing the risk of selection bias.

In the different meta-analyses, substantial heterogeneity between studies was detected. Therefore, pooled results should be viewed as an average representing a wide distribution of seroresponses. Differences in the definition and cut-off points for serological outcomes partly explained the large heterogeneity. Due to inconsistent outcome reporting across studies, we used seroresponse, a composite of seroprotection, seropositivity or seroconversion. We encourage consistency in reporting to allow for comparison between studies.

The findings from this review support the 2017 recommendations by the World Health Organization to administer an initial dose of measles vaccination at 6-months of age in areas with high incidence of HIV-infection and measles, followed by two routine doses [109]. To date, only three studies with comparison groups have evaluated immunogenicity after standard-titre measles vaccination at 6-months of age [35], [43], [61]. Future studies should evaluate serological response to early measles vaccination in HIV-infected and HEU children. In addition, there are concerns regarding long-term immunogenicity of a 2-dose schedule given early in life, as antibody titres in HIV-infected children on ART wane over time [65], [79]. Therefore, we recommend future studies on long-term waning of immunogenicity after early vaccination in HIV-infected children treated with ART.

## Contributors

EM, MCN, KKG participated in the conception, design and implementation of the study. EM and MvR performed screening and data extraction. EM did the statistical analysis. EM wrote the first draft of the report with input from MCN, MvR, KKG, DEG and SAM. All authors have approved the final manuscript.

## Declaration of Interests

MCN reports personal fees from Pfizer and non-financial support from Sanofi outside the submitted work. SAM reports grants from Medical Research Council South Africa, grants from Department Science and Technology/National Research Foundation during the conduct of the study; grants and personal fees from the Bill and Melinda Gates Foundation, grants from GSK, grants and personal fees from Sanofi, grants from Pfizer outside the submitted work. All other authors declare no competing interests.

## Appendix A Supplementary Data

Supplementary material Image 1
