# Supplementary material for: Safety and Immunogenicity of Measles Vaccination in HIV-Infected and HIV-Exposed Uninfected Children: A Systematic Review and Meta-Analysis
Source: eClinicalMedicine. 2018 Jul 2;1:28–42. doi: 10.1016/j.eclinm.2018.06.002 (PMC6537570; doi:10.1016/j.eclinm.2018.06.002)
Supplement: Supplementary file 1 — Supplementary material [file mmc1.pdf]

Appendix

Supplementary data 1: Search strategy

	**PubMed search: May 9, 2018**	**Number of results**
#1	HIV	331,300
#2	(measles AND vaccine) OR (measles AND vaccination) OR (measles AND immunization) OR (measles AND immunisation)	14,250
#3	#1 AND #2	396

	**Embase search: May 9, 2018**	**Number of results**
#1	HIV	304,410
#2	(measles AND vaccine) OR (measles AND vaccination) OR (measles AND immunization) OR (measles AND immunisation)	16,990
#3	#1 AND #2	665

	**The Cochrane Library search: May 9, 2018**	**Number of results**
#1	HIV (Word variations have been searched)	18,691
#2	(measles AND vaccine) OR (measles AND vaccination) OR (measles AND immunization) OR (measles AND immunisation)	781
#3	#1 AND #2	63

	**Cumulative Index to Nursing and Allied Health Literature (CINAHL) search: May 9, 2018**	**Number of results**
#1	HIV*	59,097
#2	(measles AND vaccine) OR (measles AND vaccination) OR (measles AND immunization) OR (measles AND immunisation) *	2,698
#3	#1 AND #2[a]	74

[a]Limited to journal articles

	**The Global Health Library (including African Index Medicus Africa and LILACS) search: May 9, 2018**	**Number of results**
#1	HIV	358,706
#2	(measles AND vaccine) OR (measles AND vaccination) OR (measles AND immunization) OR (measles AND immunisation)	17,918
#3	#1 AND #2	317

	**IndMED search: May 9, 2018**	**Number of results**
#1	HIV	2009
#2	(measles AND vaccine) OR (measles AND vaccination) OR (measles AND immunization) OR (measles AND immunisation)	89
#3	#1 AND #2	1

	**Clinical trials.gov search: May 9, 2018**	**Number of results**
#1	HIV measles (ongoing or results not published)	1[b]

[b]Ongoing study by authors of this systematic review and meta-analysis (Mutsaerts et al).

Supplementary data 2: Standardised data extraction form

Study characteristics:

1	Study ID (author, year)	Date extraction	Name of researcher performing extraction	Year of publication	Study year (start)	Country	Study setting	Primary study	Publication type	Study design	HIV-exposed/infected	Comparison group	Study population	Groups examined
2														
3														
4														

1	Study ID (author, year)	Name of vaccine	Informed consent obtained	Ethical approval obtained	Potential references from ref list	Potential references from ref list	Potential references from ref list	Immunogenicity outcome measures; I0 not reported, I1 seropositivity after vaccination reported, I2 seroconversion (seronegative before vacc, seropos after vacc), I3 seroconversion (4-fold rise in titre), I4 might be either seropositivity, seroconversion or seroprotection, I5 summary measure GMT, I6 Seroprotection	Safety outcome measures; S0 no adverse event information reported, S1 explicit reporting on adverse events, S2 serious adverse events S3 information on deaths reported	Measles outcomes: M0 no info on occurrence of clinical measles or unclear when or which group, M1 reports explicitialy on clinical measles after vacccination and numbers given per group	Information on progression of HIV-related disease reported P1
2											
3											
4											

Baseline characteristics:

1	Study ID	Age at last vaccination	Age at MV1 (months)	Stratified age MV1	Age at MV2 (months)	Stratified age MV2	Period between vaccine and serology (months) HIV infected	Period between vaccine and serology (months) HIV-uninfected	Period between vaccine and safety assessment (months)	Number of doses of measles vaccine given prior to entry
2										
3										
4										

1	Study ID	Number of HIV-exposed uninfected (HEU)	number of HIV-infected (HI)	number of HIV-unexposed uninfected	Did HIV status change during study period?	If yes, were participants included in the final analysis?	Age (median in years) of participant	Gender (% female)	Ethnicity (%)	ART use	CD4 count performed?	CD4 count of HIV infected	Variance, IQR or range of CD4 count
2													
3													
4													

Immunogenicity:

1	Study ID	Laboratory assay used for measurement of immune response	Number of vaccinations	Blood draw for serology < 6 months after vaccination	HAART	Cut-off seropositivity	Unclear if seropositive prior to vaccination are exluded	Cut-off seroprotection	Cut-off seroconversion	Proportion seropositive HEU (n)	Total	Proportion seropositive HIV-infected (n)	Total	Proportion seropositive HUU (n)	Total
2															
3															
4															

1	Study ID	Proportion seroprotected HEU (n)	Total	Proportion seroprotected HIV-infected (n)	Total	Proportion seroprotected HUU (n)	Total	Proportion seroconverted HEU (n)	Total	Proportion seroconverted HIV-infected (n)	Total	Proportion seroconverted HUU (n)	Total
2													
3													
4													

Safety:

1	Study ID	Safety reported	Comments	Number of AEs total	Number of SAEs total	Time observed for SAEs	Number of grade 1 or more AEs HEU	Total HIV-exposed	Number of grade 1 or more AEs HIV-infected	Total HIV-infected	Number of grade 1 or more AEs HUU	Total HUU	Number of SAEs HEU	Total HIV-exposed	Number of SAEs HIV-infected	Total HIV-infected	Number of SAEs HUU	Total HUU
2																		
3																		
4																		

1	Study ID	Vaccine related SAE in HEU	Vaccine related SAE in HIV-infected	Vaccine related SAE in HIV-uninfected	Type of SAEs HEU	Type of SAEs HIV-infected	Type of SAEs HUU	Duration mild AEs HEU	duration mild AEs HIV-infected	duration mild AEs HUU	Duration SAEs HEU	Duration SAEs HIV-infected	Duration SAEs HUU	Post-vaccination deaths in HIV-infected children	Vaccine related potentially life-threatening events or deaths	Time observed for deaths
2																
3																
4																

Supplementary data 3: Adapted risk of bias tool

Risk category	Low risk (=0)	High risk (=2)	Unclear risk (=1)
**Selection of study population: (selection bias)**	The individuals selected to participate are representative of the target population.	The individuals selected to participate are somewhat likely/not likely to be representative of the target population.	Not described whether individuals selected to participate in the study are likely to be representative of the target population.
	The investigators described a random component in the sequence generation process.	The investigators described a non-random component in the sequence generation process.	Insufficient information about the sequence generation process to permit judgement of 'Yes' or 'No'.
**Case-control specific:**	Case definition is adequate with independent validation.	Case definition is inadequate, e.g. record linkage or based on self-reports.	No description of the case definition.
	Consecutive or obviously representative series of cases.	Potential for selection biases.	Representativeness of cases not stated.
	Selection of controls occurred from community controls with no history of disease.	Controls are hospital controls.	There is no description of the controls.
**Cohort specific:**	Representativeness of the exposed cohort is truly representative of the population in the community.	Representativeness of the exposed cohort is somewhat representative of the population in the community.	There is no description of the derivation of the cohort.
	The non-exposed cohort is drawn from the same community as the exposed cohort.	The non-exposed cohort is drawn from a different community.	There is no description of the derivation of the non-exposed cohort.
**Cross-sectional specific:**	All the subjects were selected or recruited from the same or similar populations (including the same time period).	Subjects were selected or recruited from different populations (or in a different time period).	No description was given of the population used for subject selection or recruitment.
	Inclusion and exclusion criteria for being in the study were prespecified and applied uniformly to all participants.	Inclusion and exclusion criteria for being in the study were not prespecified or not applied uniformly to all participants.	Inclusion and exclusion criteria for being in the study are not described.
**Completeness: (attrition bias)**	No missing outcome data.	Reason for missing outcome data likely to be related to true outcome, with either imbalance in numbers or reasons for missing data across intervention groups.	Insufficient reporting of attrition/exclusions to permit judgement of 'Low-risk' or 'High-risk' (e.g. number randomised not stated, no reasons for missing data provided).
	Reasons for missing outcome data unlikely to be related to true outcome (for survival data, censoring unlikely to be introducing bias).	For dichotomous outcome data, the proportion of missing outcomes compared to observed event risk is enough to induce clinically relevant bias in intervention effect estimate.	The study did not address this outcome.
	Missing outcome data balanced in numbers across intervention groups, with similar reasons for missing data across groups.	For continuous outcome data, plausible effect size (difference in means or standardized difference in means) among missing outcomes enough to induce clinically relevant bias in observed effect size.	
	For dichotomous outcome data, the proportion of missing outcomes compared to observed event risk is not enough to have a clinically relevant impact on the intervention effect estimate.	'As-treated' analysis done with substantial departure of the intervention received from that assigned at randomisation.	

	For continuous outcome data, plausible effect size (difference in means or standardized difference in means) among missing outcomes not enough to have a clinically relevant impact on observed effect size.	Potentially inappropriate application of simple imputation.	
	Missing data have been imputed using appropriate methods.	Lost to follow-up is mentioned but there is a large size variation between the patient and control group.	
	Lost to follow-up is mentioned and is comparable between the patient and control group.		
**Case-control specific:**	Non-response rate was similar for both groups.	Non-respondents described.	Non-response rate different and no designation.
**Origin of data:**	Data was collected by researchers or by adequate personnel (midwife, research assistant etc.).	Data from database (not collected by researchers themselves).	Article does not describe where data came from.
**Cohort specific:**	Assessment of outcome occurred through independent blind assessment and/or record linkage.	Assessment of outcome occurred through self-report by participants.	No description of assessment of outcome.
**Definition of outcome:**	Article gives adequate definitions of all outcome measurements.	Article does not give adequate definitions of some outcome measurements.	Article does not give adequate definitions of all outcome measurements.
**Confounders:**	Article states that confounders (age, time period between vaccination and measurement of serological response, and CD4+ T-lymphocyte cell count if HIV-infected) were taken into account and defines these confounders.	No confounder (age, time period between vaccination and measurement of serological response, and CD4+ T-lymphocyte cell count if HIV-infected) was taken into account in the article.	Article states that some, but not all, confounders (age, time period between vaccination and measurement of serological response, and CD4+ T-lymphocyte cell count if HIV-infected) were taken into account.

Supplementary data 4: Characteristics of included studies by study design

Randomised controlled trial and cohort studies												
**Study (year)**	**Country**	**Type of study**	**Start study**	**Population**	**Groups**	**Vaccine used**	**Age at last vaccination**	**Outcomes reported**	**number of HI**	**number of HEU**	**number of HU**	**Interval between vaccination and serological testing**
Abzug 2012[1]	USA	Prospective cohort	2001	HIV-infected children 2 to <19 yr on HAART, HIV loads <30 000 copies/mL, CD4% ≥15, and ≥1 prior MMR	HI	Strain NR, MMR	2-19 yr (median 9·8 yr)	I1, I3, I5, I6, S2, S3	193	0	0	8 wks - 1·67 yr; revacc approx 7-28 days
Aurpibul 2007[2]	Thailand	Prospective cohort	2005	Perinatally HIV-infected >5 yr, nadir CD4 <15%, immune recovery>15%, for at least 3 mo on HAART, measles seronegative	HI	Schwarz, MMR	Assume at age tested negative in previous study 9·9 yr ± 2·7	I5, I6, S1, S2	51	0	0	Approx 4-24 wks
Bekker 2006[3]	Nether-lands	Prospective cohort	1997	HIV-1 infected, <18 yr, on HAART	HI	Strain NR, MMR	MV1 (n=3) at 14 mo, MV2 (n=15) at median 7·3 yr	I4, S0	59 (18 revacc)	0	0	median 48 wks (IQR 19-93 wks) (revacc)
Cagigi 2014[4]	Italy	Prospective cohort	2007	Vertically HIV-infected on HAART at children's hospital	HI	Schwarz, MMR	NR	I6, S0	32	0	0	Approximately 2·5 yr
Chandwani 2011 (& Chandwani 1998)[5,6]	USA	Randomised controlled trial	1996	Children born to HIV-infected mothers	HI, HEU	Enders' attenuated Edmonston strain, MMR	Approx 12 mo	I5, I6, S1, S2, S3	15	95	0	0 - approx 2·5 yr
Cutts 1993[7]	Zaire	Prospective cohort	1989	Infants born to HIV-infected mothers and non-HIV-infected mothers, perinatal transmission study	HI, HEU, HU	High-titre Edmonston -Zagreb strain, monovalent	Approx 6 mo (median 27 wks)	I3, S1, S2, S3	34	153	102	Approx 3 mo
Dunn 1998[8]	Europe	Prospective cohort	1985	Children born to HIV-infected mothers, European Collaborative study in 10 paediatric centres	HI, HEU	Strain NR, MMR	NR	I0, S2	17	unclear	0	NR
Farquhar 2009[9]	Kenya	Prospective cohort	2004	Previously vaccinated HIV-1 infected children before HAART initiation	HI	Strain NR, preparation NR	<1 yr	I1, S1	90	0	0	Approx 2 wks or 1 mo post-revaccination
Fernandez-Ibieta 2007[10]	Spain	Retrospectiv e cohort	1997	HIV-infected children at Pediatric Infectious Disease and Immunodeficiency Unit, 1·5-19·0 yr	HI	Strain NR, MMR	Unclear	I0, S1, S2 based on adverse event statement	68 (55 vacc)	0	0	NA
Fowlkes 2011 (& Helfand 2008)[11,12]	Malawi	Prospective cohort	2000	Children attending 14-week routine immunisation visit at health centre	HI, HEU, HU	Edmonston -Zagreb, monovalent	Approx 9 mo	I1, I6, S1, S2, S3	85	334	1327	Approx 3-15 mo

Fowlkes 2016[13]	Malawi	Prospective cohort	2000	Children enrolled in measles study who had received sMV >3 mo after their routine 9-month MV	HI, HEU, HU	Edmonston -Zagreb, monovalent	Approx 20 mo	I1, I5, I6, S2, S3	22	464 (HU)	401	<3 mo, 3-6 mo, 6-9 mo or >9 mo
Hilgartner 2001[14]	USA	Prospective cohort	1989	Children and adolescents with haemophilia	HI, HU	Strain NR, preparation NR	Min 6 mo	I5, S0	52 (24 revacc)	0	23 (10 revacc)	Approx 3-9 mo
Jain 2017[15]	India	Prospective cohort	2012	HIV-exposed infants at pediatric ART centre	HI, HEU	Edmonston -Zagreb, monovalent	6-7 mo	I1, I2, S1, S2	6	33	0	Approx 8-12 wks
Kizito 2013[16]	Uganda	Prospective cohort	2003	Pregnant women and their offspring	HI, HEU, HU	Edmonston - Zagreb/Schwarz, monovalent	Approx 9 mo	I6, S0	12	62	637	Approx 3 mo
Lepage 1992[17]	Rwanda	Prospective cohort	1988	Children born to HIV-seropositive and seronegative mothers	HI, HEU, HU	High-dose Edmonston Zagreb, monovalent	median 0·51 yr (0·48-0·84 yr)	I1, I2, I5, I6, S1, S2, S3	43	135	194	Approx 3 mo
Marczynska 2001 (substudy)[18]	Poland	Prospective cohort	NR	Children revaccinated if measles seronegative	HI	Schwarz, MMR	5-6 yr	I0, S1, S2	9	0	0	NA
McLaughlin 1988[19]	USA	Retrospective cohort	1985	Children <12 yr, vaccinated before HIV diagnosis	HI	Strain NR, monovalent or MMR	Unclear	I0, S1, S2, S3	70	0	0	NA
Melvin 2003[20]	USA	Retrospective cohort	NR	Perinatally infected children, primary immunisation series before HAART initiation, revaccination on HAART	HI	Edmonston strain, MMR	median 7 yr (range 3-14 yr)	I4, S0	18	0	0	Approx 4 wks
Moss 2007[21]	Zambia	Prospective cohort	2000	Children between 2-8 mo seeking routine childhood vaccinations	HI, HEU, HU	Edmonston -Zagreb, preparation NR	Approx 9 mo	I1, I3, I5, S1, S2, S2	66	258	117	Approx 1-6 mo
Nair 2009[22]	Zambia	Prospective cohort	2000	Children in measles vaccine study and children hospitalized for measles	HI, HU	Edmonston -Zagreb, preparation NR	Approx 9 mo	I5, S0	15	29 (HU)	0	Approx 3 mo
Nduati 2016 (& Nduati 2012)[23,24]	Kenya	Prospective cohort	2009	Children at comprehensive care and research clinic	HEU, HU	Strain NR, preparation NR	Approx 9 mo	I5, I6, S0	0	55	48	Approx 9, 12 or 15 mo
Newman 2017 (& Newman 2015)[25,26]	Kenya	Prospective cohort	2011	HIV-infected children 15 mo-12 yr	HI	Strain NR, preparation NR	Approx 9 mo	I1, S0	232	0	0	Approx 1, 12, 24 mo
Oldakowska 2001[27]	Poland	Prospective cohort	NR	HIV-infected children at pediatric infectious disease clinic	HI	Strain NR, MMR	NR	I4, S1	13	0	0	Approx 3 mo

Oldakowska 2008[28]	Poland	Prospective cohort	2001	Vertically HIV-infected children at pediatric infectious disease clinic	HI	Strain NR, preparation NR	NR	I4, S0	45	0	0	unclear
Omenda 2015[29]	Kenya	Prospective cohort	NR	HEU, HU/malaria-exposed and HU/malaria-unexposed children 0-21 mo	HEU, HU	Strain NR, preparation NR	NR	I0, S0	0	13	43 (25 malaria-exp;18 malariaunexp)	NR
Oxtoby 1989[30]	Zaire	Prospective cohort	NR	Children born to HIV-infected and HIV-uninfected mothers	HI, HEU, HU	Strain NR, preparation NR	Approx 9 mo	I2, S1, S2, S3	37	157 (385 exposed vacc)	224 (569 vacc)	Approx 12 mo
Palumbo 1992 (& Hoyt 1992)[31,32]	USA	Prospective cohort and retrospective case finding	1990	Children approx 1-10 yr	HI	Edmonston strain, MMR	Unclear	I2, S1, S2, S3	127 (92 vacc)	0	0	Approx 4 wks
Rainwater-Lovett 2013[33]	Zambia	Prospective cohort	2008	HIV-infected children 9-60 mo with documented history of MV and initiation of HAART	HI, HU (presumed)	Strain NR, preparation NR	Median 10 mo	I1, I2, S0	116	0	25	Median 11 mo
Reikie 2013[34]	South Africa	Prospective cohort	2009	Children at academic hospital	HEU, HU	Strain NR, preparation NR	Approx 18 mo	I5, I6, S0	0	27	28	Approx 3, 9, 13 mo
Seth 2016[35]	India	Prospective cohort	2011	Perinatally HIV-infected children 5-18 yr, ART>6 mo, CD4 count>15% at tertiary teaching hospital	HI	Edmonston -Zagreb, MMR	NR	I1, S1, S2	66	0	0	Approx 8-12 wks
Siberry 2015[36]	USA	Prospective cohort	2007	Perinatally HIV-infected and HEU children aged 7-15 yr at 15 centres	HI, HEU	Edmonston -Zagreb, MMR	Median 4·32 yr (IQR 4·04-5·03 yr)	I6, S0	428	221	0	median 9·8 yr (IQR 6·9-12·1 yr)
Simani 2013[37]	South Africa	Prospective cohort (archived serum samples)	2005	Children aged 6-12 wks	HI, HEU, HU	Schwarz, monovalent	Mean 67·8 wks ± 4·4	I1, I5, I6, S0	297	116	115	28 wks post MV1, 2 and 41 wks post MV2
Succi 2018[38]	Latin America	Prospective cohort	2002	Perinatally HIV-infected children and HEU children <4 yrs at 15 centres	HI, HEU	Strain NR, preparation NR	Approx 12 mo	I1, I5, S0	96	51	0	median 1037 days (2·8 yrs)
Sudfeld 2013[39]	Tanzania	Prospective cohort	2005	Children enrolled in trial on multivitamin supplementation	HI, HEU	Edmonston -Zagreb, preparation NR	Approx 9 mo (8·5-12 mo)	I1, I5, S0	35	201	0	Approx 3-9·5 mo
Takano 2003[40]	Brazil	Prospective cohort	NR	Children at paediatric HIV clinic	HI, HU	Strain NR, MMR	Min 1 yr	I1, I5, S0	70 (12 revacc)	0	69	Approx 1-3 mo
Thaithumyanon 2000[41]	Thailand	Prospective cohort	NR	Children born to HIV-infected mothers	HI, HEU	Schwarz, monovalent	Approx 9 mo	I2, I5, S1, S2, S3	16	14	3	Approx 12 wks

Cross-sectional studies, prospective cohort studies/cross-sectional, retrospective cohort studies/cross-sectional												
**Study**	**Country**	**Type of study**	**Start study**	**Population**	**Groups**	**Vaccine used**	**Age at last vaccination**	**Outcomes reported***	**number of HI**	**number of HEU**	**number of HU**	**Interval between vaccination and serology**
al-Attar 1995[42]	USA	Retrospective cohort / cross-sectional	1986	Children at HIV clinic, born to HIV-positive mothers or to mothers with high risk of being HIV-infected	HI, HEU	Strain NR, preparation NR	1·2-2·3 yr (median 1·3 yr)	I4, I5, S0	40	16 (HU)	0	1 mo - 6·7 yr (mean 1·6 yr)
Arpadi 1996 (& Arpadi 1992)[43,44]	USA	Cross-sectional	1991	Perinatally HIV-infected children 9-168 mo	HI	strain NR, monovalent or MMR	Unclear	I1, S0	81	0	0	1-155 mo (median 6 mo)
Aurpibul 2006[45]	Thailand	Cross-sectional	2005	Perinatally HIV-infected children >5 yr, nadir CD4% <15%, immune recovery>15% for at least 3 mo on HAART	HI	Strain NR, preparation NR	Unclear	I5, I6, S0	93	0	0	79·6 ±38·6 mo
Berkelhamer 2001[46]	USA	Retrospective cohort / cross-sectional	1999	Perinatally HIV-infected <12 yr, untreated, non-HAART, or HAART-regimen receiving medical care at children's hospital	HI	Strain NR, MMR	mean 6 yr (3·9-11 yr)	I6, S0	28	0	0	HAART mean 2·3 mo (range 1-4 mo), non-HAART mean 3·7 mo (range 1-9 mo)
Brena 1993[47]	USA	Retrospective cohort / cross-sectional	NR	HIV-infected children and children born to HIV-infected mothers who seroreverted to negative	HI, HEU	Strain NR, MMR	median 1·3 yr (1·2-3 yr)	I1, I5, S0	20	13	0	median 2 mo (range 1-42 mo)
Cardemil 2016a[48]	Namibia	Cross-sectional	2008	Pregnant women aged 15-49 yr at first ANC visit, not referred from other health facility	HI, HU	Strain NR, preparation NR	NR	I4, S0	4 (aged 15-19 yr)	0	332 (aged 15-19 yr)	NR
Cardemil 2016b[48]	Namibia	Cross-sectional	2010	Pregnant women aged 15-49 yr at first ANC visit, not referred from other health facility	HI, HU	Strain NR, preparation NR	NR	I4, S0	24 (aged 15-19 yr)	335 (aged 15-19 yr)	NR	NR
Echeverria Lecuona 1996[49]	Spain	Retrospective cohort / cross-sectional	NR	Children born to HIV-infected mothers at study hospital	HI, HEU	Strain NR, MMR	Approx 12 mo	I1, S1, S2 based on adverse event statement	14 (10 vacc)	30	0	Approx 1-2 yr
Fitter 2013[50]	Haiti	Cross-sectional	2012	Pregnant women aged 15-39 yr in national antenatal HIV sentinel serosurvey	HI	Strain NR, preparation NR	Unclear	I1, I6, S0	184 (aged 15-19 yr)	0	0	NR
Frenkel 1994 (& Frenkel 1992)[51,52]	USA	Prospective cohort / cross-sectional	NR	HIV-infected symptomatic children with documented history of MMR	HI	Strain NR, MMR	Unclear	I4, S1, S2	10	0	0	median 13 mo (range 1-130 mo)

Lindgren-Alves 2001[53]	Brazil	Retrospective cohort / cross-sectional	1995	Perinatally infected children at teaching hospital HIV-centre	HI, HU	Strain NR, preparation NR	Unclear	I4, I5, S0	21	0	29	Mean 29·4 mo ±31·9 mo
Lowther 2009[54]	Zambia	Cross-sectional	2006	Children 9 mo-5 yr from randomly selected households	HI, HU	Strain NR, preparation NR	Approx 9 mo	I4, S0	54	0	796	Unclear
Lyamuya 1999[55]	Tanzania	Cross-sectional	1994	Children 18 mo-5 yr attending mother and child health clinics	HI, HIV-uninfected	Schwarz, preparation NR	Approx 9 mo	I5, I6, S0	9	0	663	Mean 26·1 mo
Marczynska 2001[18]	Poland	Retrospective cohort / cross-sectional	NR	HIV-infected children on HAART; matched controls	HI, HIV-uninfected	Schwarz, monovalent or MMR	Unclear	I1, S0	19	0	19	Mean 3·1 yr (range 3 mo-13 yr)
Molyneaux 1993[56]	UK	Retrospective cohort / cross-sectional	NR	Children in HIV perinatal transmission study aged >1 yr	HI, HEU	Strain NR, monovalent or MMR	Min 1 yr	I1, S1, S2	11 (9 vacc)	70 (61 vacc)	0	Approx 3-9 mo
Morris 2015[57]	USA	Prospective cohort / cross-sectional	2011	Individuals with perinatally-acquired HIV aged 13-26 yr at HIV-clinic	HI	Strain NR, MMR	NR	I4, S0	34	0	0	non-immune median 13·5 mo (range 10·8-15 mo), immune: median 7·5 mo (range 6-9 mo)
Myers 2009[58]	Switzerland	Prospective cohort / cross-sectional	NR	Children part of mother and child HIV cohort study	HI	Strain NR, MMR	Unclear	I4, S0	87	0	0	NR
Ndikuyeze 1987[59]	Rwanda	Cross-sectional	1985	Children 8-19 mo	HI	Strain NR, preparation NR	Approx 8-19 mo	I0, S1, S2	3	492 (HU)	0	NR
Newman 2014[60]	Kenya	Cross-sectional	2011	HIV-infected children 15 mo-12 yr	HI	Strain NR, preparation NR	Approx 9 mo	I4, S0	232	0	0	NR
Oshitani 1996[61]	Zambia	Cross-sectional	1993	Children 9-59 mo with measles in University teaching hospital	HI, HU	Biken-cam, preparation NR	Approx 9 mo	I0, S3	68 (37 vacc)	0	288 (111 vacc)	NR
Pensieroso 2009[62]	Italy	Cross-sectional	2007	Vertically HIV-infected children at children's hospital; age matched healthy controls	HI, HU	Schwarz, MMR	NR	I2, I6, S0	70	0	50	Mean 4·7 yr
Polonsky 2015 (& Polonsky 2015)[63,64]	Malawi	Cross-sectional	2012	Individuals presenting for HIV testing at district hospital	HI, HU	Strain NR, preparation NR	NR	I4, I5, I6, S0	86 (aged 18 mo-17 yr)	0	55 (aged 18 mo - 17 yr)	NR
Rosso 2011[65]	Italy	Retrospective cohort / cross-sectional	NR	Perinatally HIV-infected adolescents	HI	Strain NR, MMR	NR	I4, S0	39	0	0	NR

Rowson 2015[66]	UK	Cross-sectional	NR	Previously vaccinated HIV-infected children	HI	Strain NR, preparation NR	NR	I4, S0	224	0	0	Approx 6-12 wks
Rudy 1994a[67]	USA	Unclear	1990	Children at immunology clinic, vaccinated < 12 mo	HI, HEU	Strain NR, monovalent	6-11 mo	I4, S1, S2	13	22	0	Approx 1-3 mo
Rudy 1994b[67]	USA	Unclear	1990	Children at immunology clinic, vaccinated ≥12 mo	HI, HEU	Strain NR, MMR	12-15 mo	I4, S1, S2	12	14	0	Approx 1·0-3·0 mo
Ruel 2008[68]	Uganda	Cross-sectional	2005	HIV-infected children 1-10 yr at paediatric HIV clinic	HI	strain NR, preparation NR	NR	I0, S0, P1	300 (11 vacc and lab test)	0	0	NA
Singh 2013[69]	UK	Retrospective cohort / cross-sectional	NR	HIV-infected children at paediatric HIV centre	HI	strain NR, MMR	NR	I4, S0	80	0	0	NR
Sticchi 2015[70]	Italy	Cross-sectional	NR	Perinatally HIV-infected children and adults at regional reference hospital	HI	strain NR, MMR	NR	I4, S0	39	0	0	NR
Sutcliffe 2016[71]	Zambia	Cross-sectional	2009	HIV-infected not on ART and HIV-infected youth receiving ART, HIV-uninfected youth (5-15 yr) in study of malaria and HIV	HI, HU	strain NR, preparation NR	Unclear	I4 (seroprevalence), S0	272	0	617	NR
Tejiokem 2007[72]	Cameroon, Central African Republic	Cross-sectional	2004	Children at 4 paediatric care centres between 18-36 mo	HI, HEU	strain NR, preparation NR	9 mo-1·3 yr	I1, I5, S0	51	78	0	Median 12·8 mo (90% range; 3·3-26·1 months)
Waibale 1999[73]	Uganda	Retrospective cohort / cross-sectional	1995	Children at paediatric and HIV clinic	HI, HEU	Strain NR, monovalent	Median 9·4 mo (5·2 - 25·8 mo)	I1, I5, S0	50	193	0	Median 14.0 mo (2·7 - 30·8 mo)
Walter 1994[74]	USA	Retrospective cohort / cross-sectional	1992	HIV-infected or born to HIV-infected mother at paediatric infectious disease clinic ≥15 mo	HI, HEU	Strain NR, MMR	Mean 20·4 month (±10·2 mo)	I4, I5, S0	35	49	0	mean 3·1, 13·3 and 43·6 mo
**Other study designs**												
**Study**	**Country**	**Type of study**	**Start study**	**Population**	**Groups**	**Vaccine used**	**Age at last vaccination**	**Outcomes reported**	**number of HI**	**number of HEU**	**number of HU**	**Interval between vaccination and serology**
Brunell 1995a[75]	USA	Unclear	1980	Perinatally HIV-infected infants; normal babies	HI, HU	Strain NR, MMR/MMRV	median 15 mo (range 8-26 mo)	I1, I5, S0	9	0	21	median 7 mo (range 2 - 29 mo)
Brunell 1995b[75]	USA	Unclear	1980	Children who received HIV-infected blood or blood	HI, HU	Strain NR, MMR	14-42 mo	I5, S0	17	0	49	median 29 mo (range 3 wks - 127 mo)

				products,1·5-9 yr; normal children								
Dhesi 2012[76]	UK	Retrospective audit	NR	HIV-positive children 4-16 yr at University hospital	HI	Strain NR, MMR	NR	I4, S0	19	0	0	NR
Embree 1989[77]	Kenya	Unclear	NR	Perinatal HIV-transmission study	HI, HEU	Strain NR, preparation NR	Unclear	I4, S1, S2 based on adverse event statement	61	98	0	Unclear
Goon 2001[78]	UK	Case report	NR	HIV-infected child	HI	Edmonston strain, monovalent	1·2 yr	I0, S1, S2, S3	1	0	0	NA
Ramon-Garcia 1995[79]	Mexico	Case report	1989	HIV-infected children during measles outbreak	HI	Strain NR, preparation NR	Approx 12 mo	I0, S2, S3	2	0	0	NA

Abbreviations: ANC, antenatal care; exp, exposed; ART, antiretroviral therapy; HEU, HIV-exposed uninfected; HI, HIV-infected; HU, HIV-unexposed; HU, HIV-unexposed; mo, months of age; MV, measles vaccination; MMR, measles, mumps, rubella vaccine; MMRV, measles, mumps, rubella, varicella vaccine; NA, not applicable; NR, not reported; revacc, revaccinated; sMV, supplemental measles vaccination; unexp, unexposed; vacc, vaccinated; yr, years of age;

*I immunogenicity outcomes: I0, immunogenicity not reported; I1, seropositivity after vaccination reported; I2, seroconversion (seronegative before vaccination, seropositive after vaccination) reported; I3, seroconversion (4-fold rise in titre) reported; I4, measure which might be either seropositivity, seroconversion or seroprotection after vaccination is reported; I5, summary immunological measure (e.g. geometric mean titre) reported; I6, seroprotection after vaccination reported;

*S Safety outcomes: S0, no adverse event information reported; S1, explicit reporting on adverse events; S2, explicit reporting on serious adverse events; S3, reporting on deaths.

Supplementary data 5:

Supplementary data 5.1: Descriptive analysis of studies reporting seroresponses after measles vaccination in HIV-infected children by dose and age at vaccination

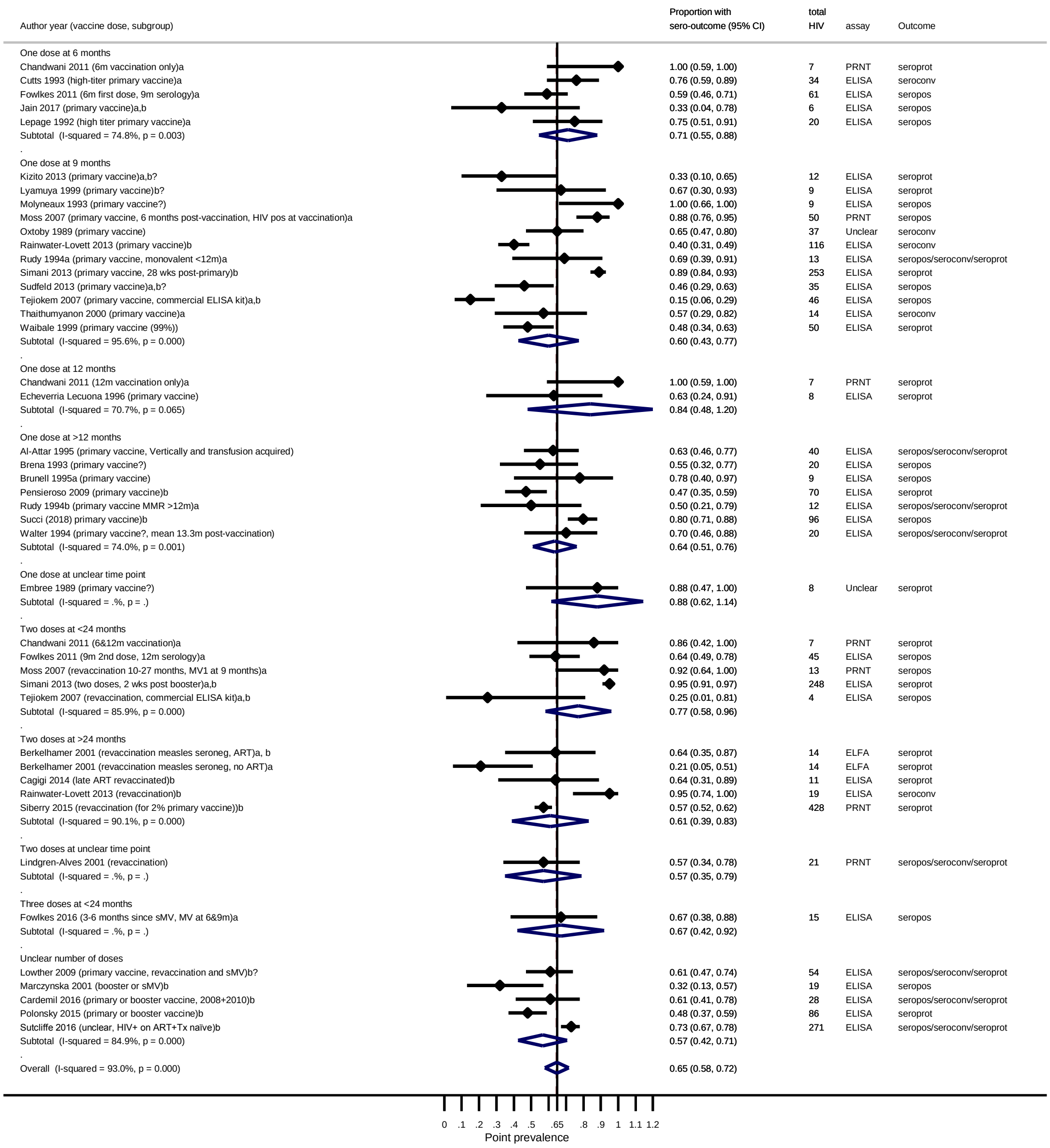

Supplementary data 5.2: Descriptive analysis of studies reporting seroresponses after measles vaccination in HEU children by dose and age at vaccination

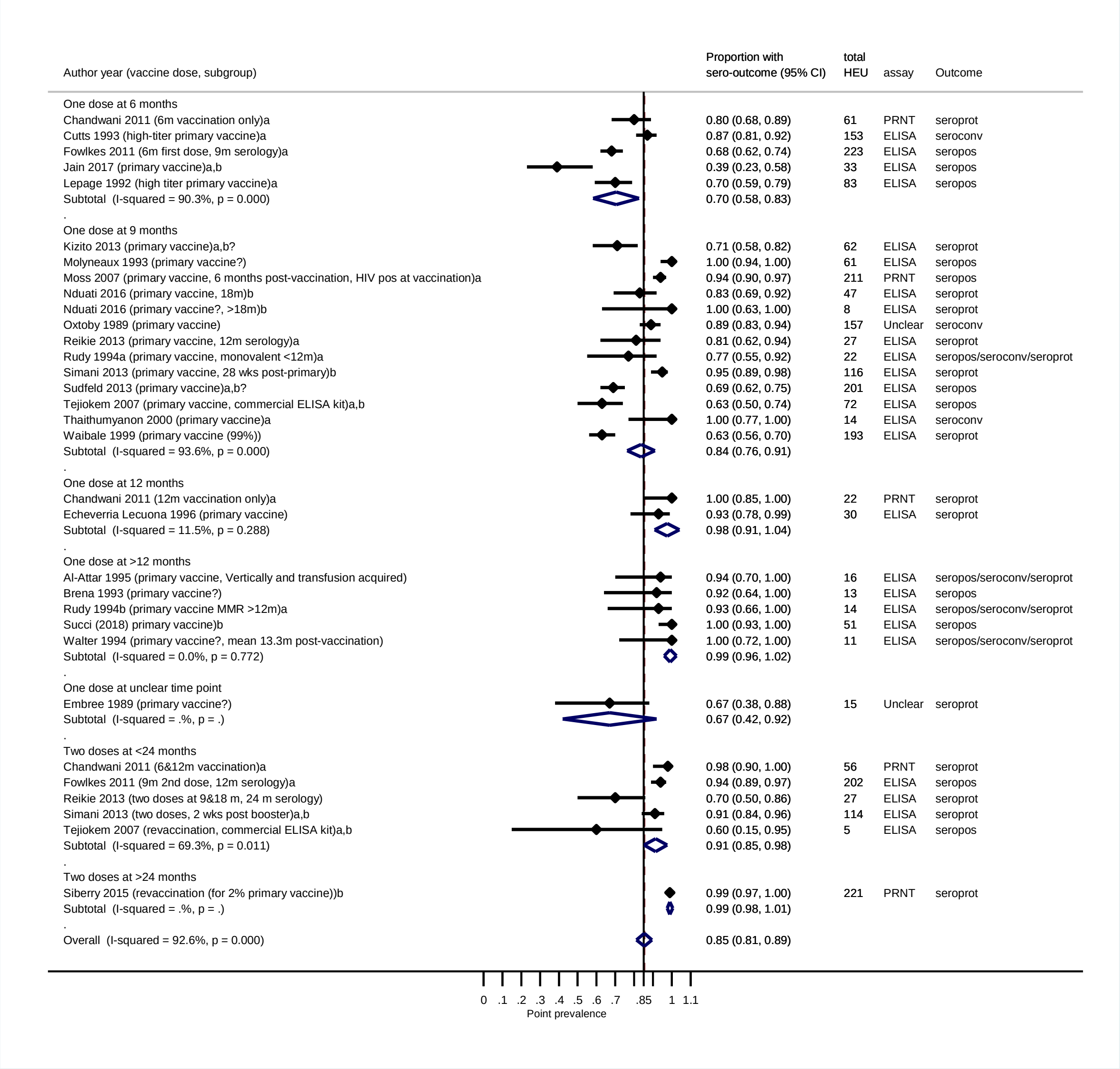

Supplementary data 5.3: Descriptive analysis of studies reporting seroresponses after measles vaccination in HIV-unexposed children by dose and age at vaccination

Author year (vaccine dose, subgroup)	Proportion with sero-outcome (95% CI)	total HU	assay	Outcome
One dose at 6 months				
Cutts 1993 (high-titer primary vaccine)a	0.83 (0.75, 0.90)	102	ELISA	seroconv
Fowlkes 2011 (6m first dose, 9m serology)a	0.62 (0.57, 0.66)	467	ELISA	seropos
Lepage 1992 (high titer primary vaccine)a	0.52 (0.41, 0.63)	87	ELISA	seropos
Subtotal (I-squared = 93.1%, p = 0.000)	0.66 (0.50, 0.82)			
.				
One dose at 9 months				
Kizito 2013 (primary vaccine)a,b?	0.76 (0.72, 0.79)	637	ELISA	seroprot
Lyamuya 1999 (primary vaccine)b?	0.93 (0.91, 0.95)	663	ELISA	seroprot
Moss 2007 (primary vaccine, 6 months post-vaccination, HIV pos at vaccination)a	0.94 (0.87, 0.98)	98	PRNT	seropos
Nduati 2016 (primary vaccine, 18m)b	0.95 (0.75, 1.00)	20	ELISA	seroprot
Nduati 2016 (primary vaccine?, >18m)b	0.93 (0.76, 0.99)	28	ELISA	seroprot
Oxtoby 1989 (primary vaccine)	0.89 (0.84, 0.93)	224	Unclear	seroconv
Rainwater-Lovett 2013 (primary vaccine)b	0.75 (0.43, 0.95)	12	ELISA	seroconv
Reikie 2013 (primary vaccine, 12m serology)a	0.71 (0.51, 0.87)	28	ELISA	seroprot
Simani 2013 (primary vaccine, 28 wks post-primary)b	0.91 (0.84, 0.96)	112	ELISA	seroprot
Subtotal (I-squared = 89.8%, p = 0.000)	0.88 (0.82, 0.94)			
.				
One dose at >12 months				
Brunell 1995a (primary vaccine)	1.00 (0.84, 1.00)	21	ELISA	seropos
Pensieroso 2009 (primary vaccine)b	1.00 (0.93, 1.00)	50	ELISA	seroprot
Subtotal (I-squared = 0.0%, p = 1.000)	1.00 (0.97, 1.03)			
.				
Two doses at <24 months				
Fowlkes 2011 (9m 2nd dose, 12m serology)a	0.92 (0.89, 0.95)	417	ELISA	seropos
Moss 2007 (revaccination 10-27 months, MV1 at 9 months)a	0.97 (0.91, 0.99)	115	PRNT	seropos
Reikie 2013 (two doses at 9&18 m, 24 m serology)	0.48 (0.29, 0.68)	27	ELISA	seroprot
Simani 2013 (two doses, 2 wks post booster)a,b	0.97 (0.91, 0.99)	115	ELISA	seroprot
Subtotal (I-squared = 89.0%, p = 0.000)	0.91 (0.84, 0.98)			
.				
Two doses at >24 months				
Rainwater-Lovett 2013 (revaccination)b	1.00 (0.75, 1.00)	13	ELISA	seroconv
Subtotal (I-squared = .%, p = .)	1.00 (0.88, 1.13)			
.				
Two doses at unclear time point				
Lindgren-Alves 2001 (revaccination)	1.00 (0.88, 1.00)	29	PRNT	seropos/seroconv/seroprot
Subtotal (I-squared = .%, p = .)	1.00 (0.94, 1.06)			
.				
Three doses at <24 months				
Fowlkes 2016 (3-6 months since sMV, MV at 6&9m)a	0.96 (0.92, 0.98)	268	ELISA	seropos
Subtotal (I-squared = .%, p = .)	0.96 (0.93, 0.99)			
.				
Unclear number of doses				
Lowther 2009 (primary vaccine, revaccination and sMV)b?	0.71 (0.68, 0.74)	796	ELISA	seropos/seroconv/seroprot
Marczynska 2001 (booster or sMV)b	1.00 (0.82, 1.00)	19	ELISA	seropos
Cardemil 2016 (primary or booster vaccine, 2008+2010)b	0.78 (0.74, 0.81)	666	ELISA	seropos/seroconv/seroprot
Polonsky 2015 (primary or booster vaccine)b	0.76 (0.63, 0.87)	55	ELISA	seroprot
Sutcliffe 2016 (unclear, HIV+ on ART+Tx naïve)b	0.93 (0.90, 0.95)	562	ELISA	seropos/seroconv/seroprot
Subtotal (I-squared = 97.3%, p = 0.000)	0.84 (0.72, 0.95)			
.				
Overall (I-squared = 95.8%, p = 0.000)	0.87 (0.82, 0.91)			

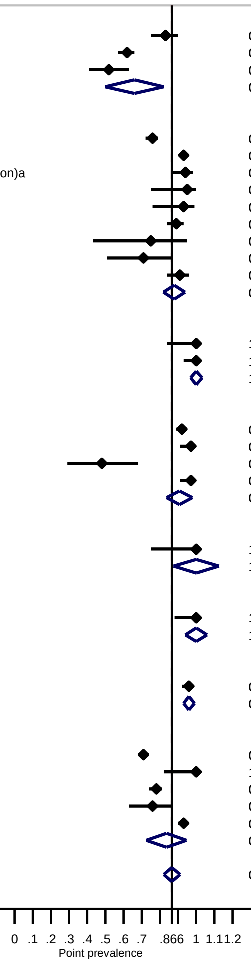

Abbreviations: ART, antiretroviral therapy; ELISA, enzyme-linked immunosorbent assay; HEU, HIV-exposed uninfected; HU, HIV-unexposed; MV, measles vaccination; MMR, measles, mumps, rubella vaccine; PRNT, plaque reduction neutralisation test; sMV, supplemental measles vaccination;

a: studies where blood was drawn for measles serology within six months after vaccination;

b: studies where children received antiretroviral therapy;

b?: studies where it is not clear if children received antiretroviral therapy.

Supplementary data 6: Subgroup analyses for immune response post-primary vaccination

RR (95% CI)	HIV vs HIV-unexposed primary vaccination	HIV vs HEU primary vaccination	HEU vs HIV-unexposed primary vaccination
Outcome in main analysis	Overall: **0·74 (0·61-0·90)**; n=9<br>6 months: 0·96 (0·77-1·19)<br>9 months: **0·79 (0·65-0·95)**<br>>12 months: **0·59 (0·37-0·95)**	Overall: **0·78 (0·69-0·88)**; n=21<br>6 months: 1·00 (0·73-1·37)<br>9 months: **0·73 (0·59-0·89)**<br>**12 months: 0·72 (0·62-0·84)**<br>>12 months: **0·66 (0·55-0·78)**<br>Unclear age: 1·31 (0·84-2·05)	Overall: 1·03 (0·98-1·07); n=7<br>6 months: 1·11 (0·99-1·24)<br>9 months: 1·00 (0·96-1·04)
Limited to studies that reported on seroprotection	Overall: 0·64 (0·36-1·14); n=4<br>9 months: 0·74 (0·44-1·24)<br>>12 month: **0·48 (0·37-0·61)**	Overall: 0·92 (0·74-1·15); n=7<br>6 months: 1·17 (0·94-1·46)<br>9 months: 0·78 (0·52-1·16)<br>12 months: 0·67 (0·39-1·16)<br>Unclear age: 1·31 (0·84-2·05)	Overall: 0·99 (0·92-1·07); n=4<br>9 months: 0·99 (0·92-1·07)
Limited to studies that reported on serology within 3 months after primary vaccination	Overall: 0·71 (0·33-1·55); n=2<br>6 months: 0·96 (0·77-1·19)<br>9 months: **0·44 (0·20-0·98)**	Overall: 0·79 (0·60-1·04); n=8<br>6 months: 1·00 (0·73-1·37)<br>9 months: **0·68 (0·46-0·99)**<br>>12 months: **0·54 (0·30-0·97)**	Overall: 1·06 (0·97-1·16); n=3<br>6 months: 1·11 (0·99-1·24)<br>9 months: 0·98 (0·84-1·13)
Limited to studies that reported on serology within 6 months after primary vaccination	Overall: 0·90 (0·73-1·11); n=3<br>6 months: 0·96 (0·77-1·19)<br>9 months: 0·68 (0·23-1·96)	Overall: **0·81 (0·67-0·99)**; n=10<br>6 months: 1·00 (0·73-1·37)<br>9 months: 0·74 (0·54-1·01)<br>>12 months: **0·54 (0·30-0·97)**	Overall: 1·04 (0·97-1·11); n=4<br>6 months: 1·11 (0·99-1·24)<br>9 months: 0·99 (0·92-1·06)
Limited to studies administering primary vaccination ≤12 months of age	Overall: **0·83 (0·71-0·9)**; n=7<br>6 months: 0·96 (0·77-1·19)<br>9 months: **0·79 (0·65-0·95)**	Overall: **0·79 (0·67-0·92)**; n=15<br>6 months: 1·00 (0·73-1·37)<br>9 months: **0·73 (0·59-0·89)**<br>12 months: 0·67 (0·39-1·16)	No difference
Limited to studies administering ART	Overall: 0·63 (0·34-1·19); n=3<br>9 months: 0·74 (0·40-1·38)<br>>12 months: **0·48 (0·37-0·61)**	Overall: 0·74 (0·54-1·00); n=4<br>6 months: 0·85 (0·25-2·83)<br>9 months: 0·49 (0·06-3·91)<br>**>12 months: 0·81 (0·73-0·90)**	NA
Excluding studies with assumed primary vaccination dose*	No difference	Overall: **0·78 (0·68-0·88)**; n=17<br>6 months: 1·00 (0·73-1·37)<br>9 months: **0·73 (0·59-0·89)**<br>12 months: 0·67 (0·39-1·16)<br>>12 months: **0·72 (0·58-0·90)**	Overall: 1·03 (0·98-1·07); n=7<br>6 months: 1·11 (0·99-1·24)<br>9 months: 1·00 (0·96-1·04)
Limited to studies with known age at vaccination	No difference	Overall: **0·76 (0·68-0·86)**; n=20<br>6 months: 1·00 (0·73-1·37)<br>9 months: **0·73 (0·59-0·89)**<br>12 months: 0·67 (0·39-1·16)<br>>12 months: 0·72 (0·62-0·84)	No difference
Limited to studies that report the first time point after vaccination	No difference	Overall: **0·78 (0·69-0·89)**; n=20<br>6 months: 1·00 (0·73-1·37)<br>9 months: **0·73 (0·59-0·89)**<br>12 months: 0·67 (0·39-1·16)<br>**>12 months: 0·70 (0·57-0·86)**<br>Unclear age: 1·31 (0·84-2·05)	No difference

Abbreviations: ART, antiretroviral therapy; CI, confidence interval; HIV, HIV-infected; HEU, HIV-exposed uninfected; NA, not applicable; No difference, if result of the sensitivity analysis is exactly the same as the main analysis; RR; risk ratio;

*Studies that did not explicitly mention that primary vaccine was administered, but where this was assumed based on the context of the paper.
Results with a significant p-value are marked in bold.

Supplementary data 7: Subgroup analyses for immune responses post-booster vaccination

RR (95% CI)	HIV vs HIV-unexposed booster vaccination	HIV vs HEU booster vaccination	HEU vs HIV-unexposed booster vaccination
Outcome in main analysis	Overall: 0·84 (0·68-1·04) ; n=5<br>≤ 24 months: 0·88 (0·68-1·14)<br>> 24 months: 0·96 (0·82-1·13)<br>Unclear age: **0·58 (0·40-0·83)**	Overall: 0·75 (0·50-1·13); n=5<br>≤ 24 months: 0·84 (0·59-1·19)<br>> 24 months: **0·58 (0·53-0·63)**	Overall: 1·00 (0·91-1·09); n=3<br>≤ 24 months: 1·00 (0·91-1·09)
Limited to studies that reported on seroprotection	Overall: 0·98 (0·94-1·03); n=1<br>≤ 24 months: 0·98 (0·94-1·03)	Overall: 0·80 (0·48-1·33); n=3<br>≤ 24 months: 1·02 (0·91-1·14)<br>> 24 months: **0·58 (0·53-0·63)**	Overall: 1·13 (0·67-1·92); n=2<br>≤ 24 months: 1·13 (0·67-1·92)
Limited to studies that reported on serology within 3 months after booster vaccination	Overall: 0·83 (0·49-1·43); n=2<br>≤ 24 months: 0·83 (0·49-1·43)	Overall: 0·86 (0·61-1·22); n=3<br>≤ 24 months: 0·86 (0·61-1·22)	Overall: 0·98 (0·92-1·05); n=2<br>≤ 24 months: 0·98 (0·92-1·05)
Limited to studies that reported on serology within 6 months after booster vaccination	Overall: 0·88 (0·68-1·14); n=3<br>≤ 24 months: 0·88 (0·68-1·14)	Overall: 0·86 (0·61-1·22); n=3<br>≤ 24 months: 0·86 (0·61-1·22)	No difference
Limited to studies administering booster vaccination ≤24 months of age	Overall: 0·88 (0·68-1·14); n=3<br>≤ 24 months: 0·88 (0·68-1·14)	Overall: 0·84 (0·59-1·19); n=4<br>≤ 24 months: 0·84 (0·59-1·19)	No difference
Limited to studies administering ART	Overall: 0·98 (0·94-1·02); n=2<br>≤ 24 months: 0·98 (0·94-1·03)<br>>24 months: 0·96 (0·82-1·13)	Overall: 0·73 (0·40-1·35); n=3<br>≤ 24 months: 1·02 (0·79-1·32)<br>> 24 months: 0·84 (0·59-1·19)	NA
Limited to studies with known age at vaccination	Overall: 0·90 (0·76-1·08); n=4<br>≤ 24 months: 0·88 (0·68-1·14)<br>> 24 months: 0·96 (0·82-1·13)	No difference	No difference

Abbreviations: ART, antiretroviral therapy; CI, confidence interval; HIV, HIV-infected; HEU, HIV-exposed uninfected; NA; not applicable; No difference, if result of the sensitivity analysis is exactly the same as the main analysis; RR; risk ratio;

Results with a significant p-value are marked in bold.

Supplementary data 8:

Supplementary data 8.1: Meta-analyses comparing HIV-infected and HIV-unexposed children after primary measles vaccination stratified by (A) serological outcome, (B) serological assay to measure antibody response, (C) administration of antiretroviral therapy, (D) study design, and (E) time interval between vaccination and measurement of serology within 6 months

(A)

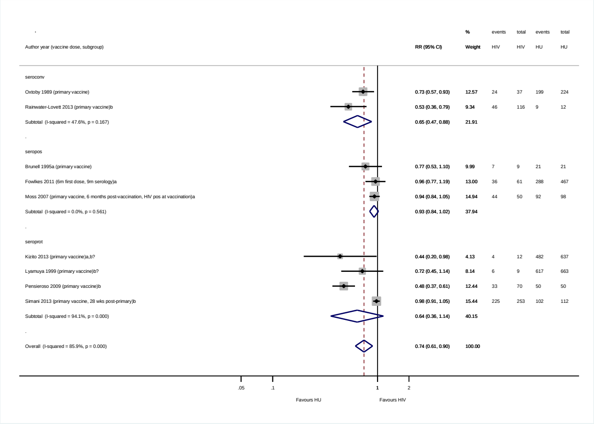

(B)

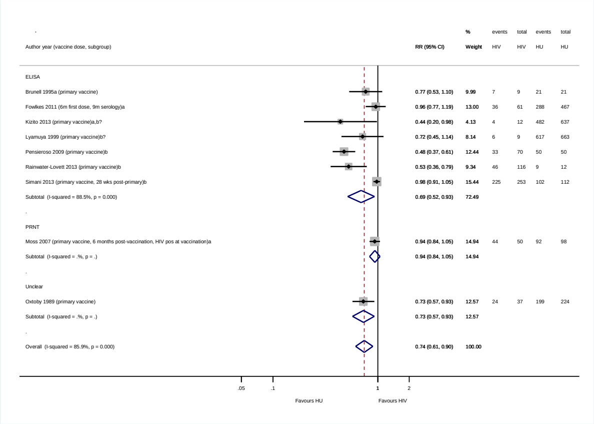

(C)

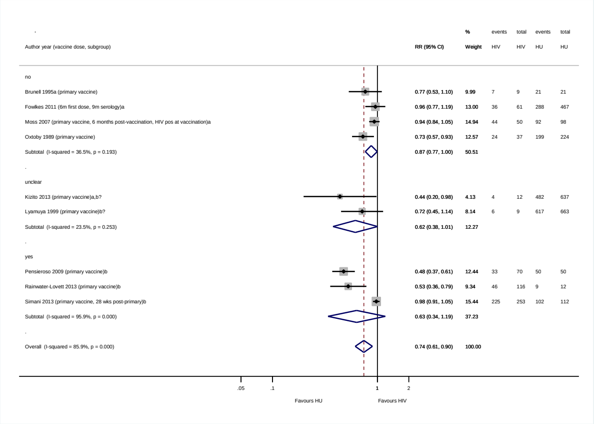

(D)

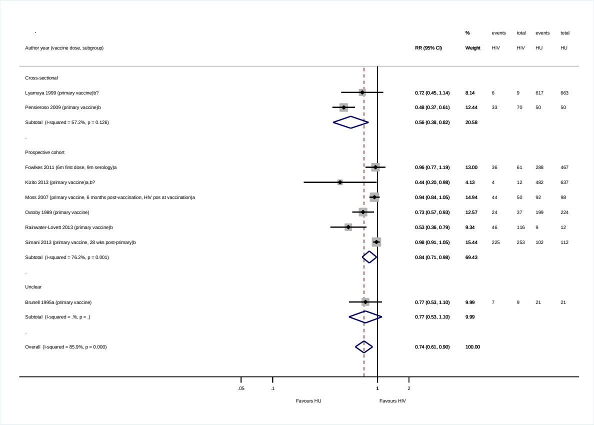

(E)

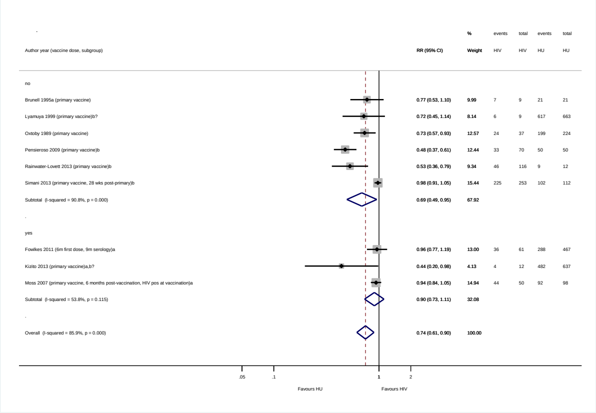

Supplementary data 8.2: Meta-analyses comparing HIV-infected and HIV-unexposed children after booster measles vaccination stratified by (A) serological outcome, (B) serological assay to measure antibody response, (C) administration of antiretroviral therapy, (D) study design, and (E) time interval between vaccination and measurement of serology within 6 months

(A)

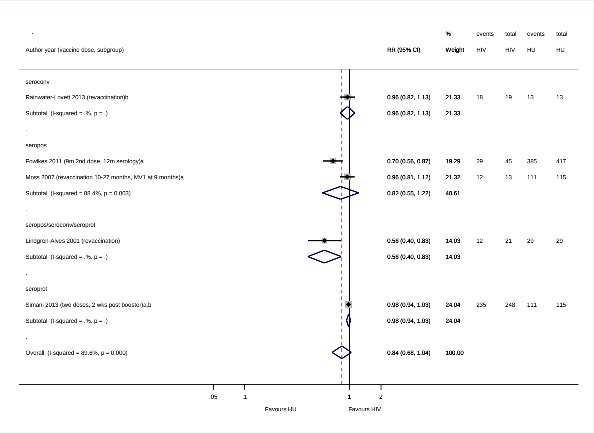

(B)

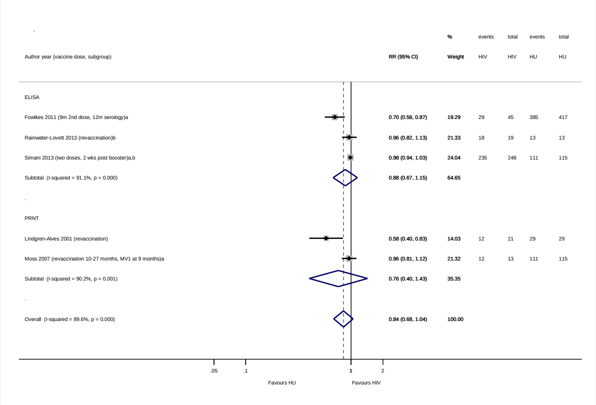

(C)

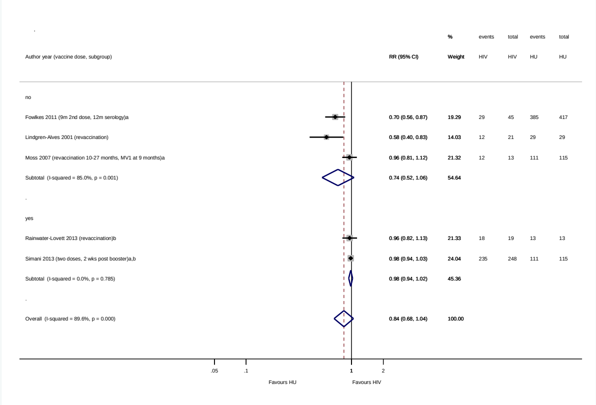

(D)

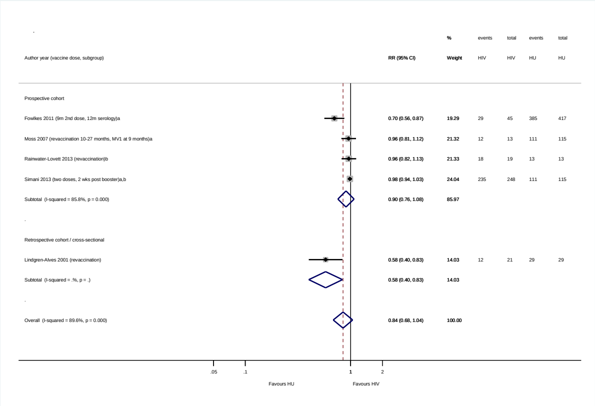

(E)

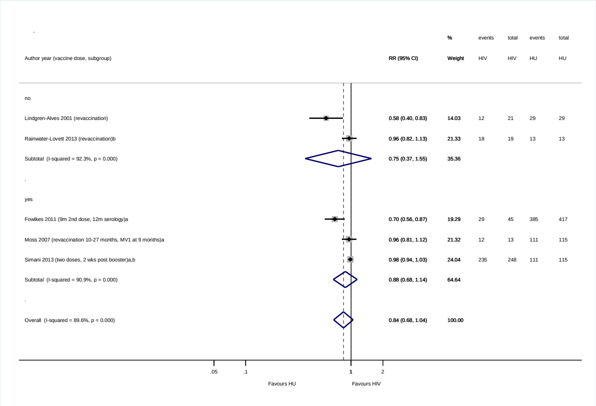

Supplementary data 8.3: Meta-analyses comparing HIV-infected and HIV-exposed uninfected children after primary measles vaccination stratified by (A) serological outcome, (B) serological assay to measure antibody response, (C) administration of antiretroviral therapy, (D) study design, and (E) time interval between vaccination and measurement of serology within 6 months

(A)

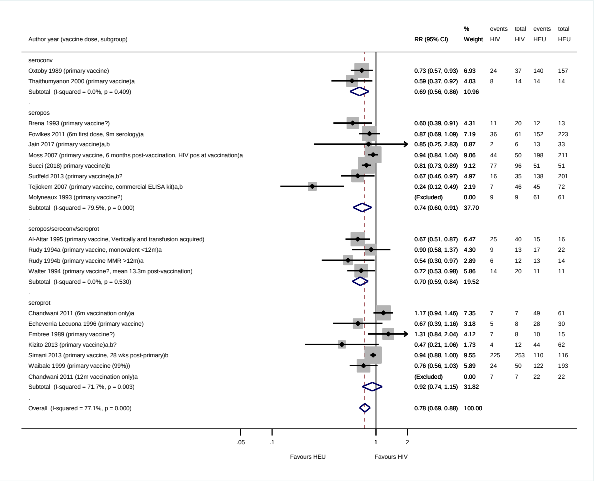

(B)

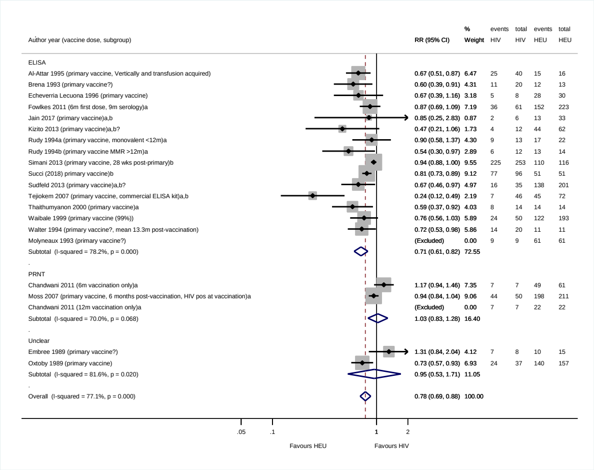

(C)

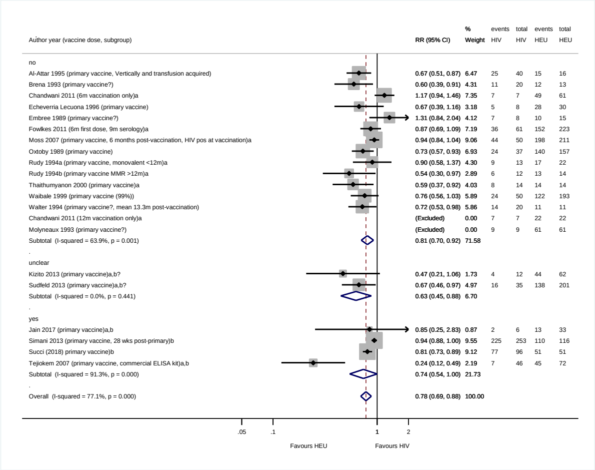

(D)

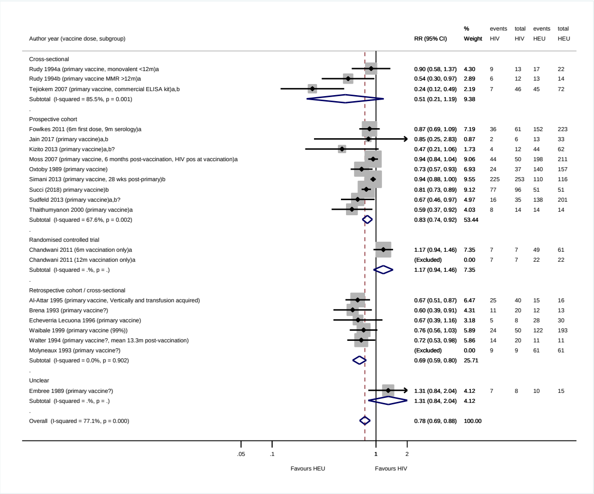

(E)

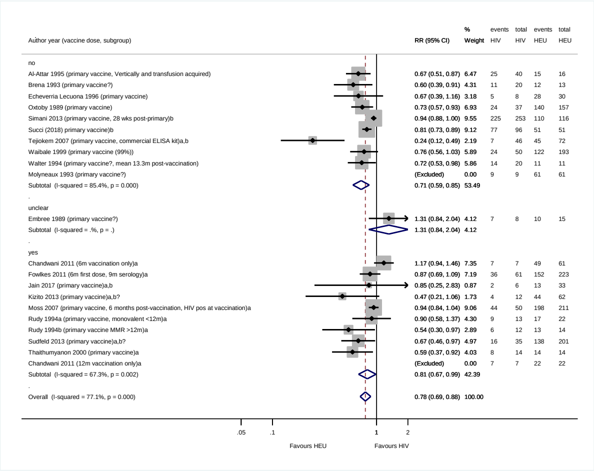

Supplementary data 8.4: Meta-analyses comparing HIV-infected and HIV-exposed uninfected children after booster measles vaccination stratified by (A) serological outcome, (B) serological assay to measure antibody response, (C) administration of antiretroviral therapy, (D) study design, and (E) time interval between vaccination and measurement of serology within 6 months

(A)

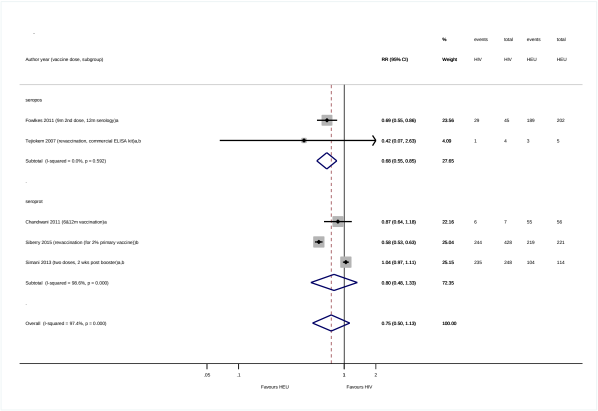

(B)

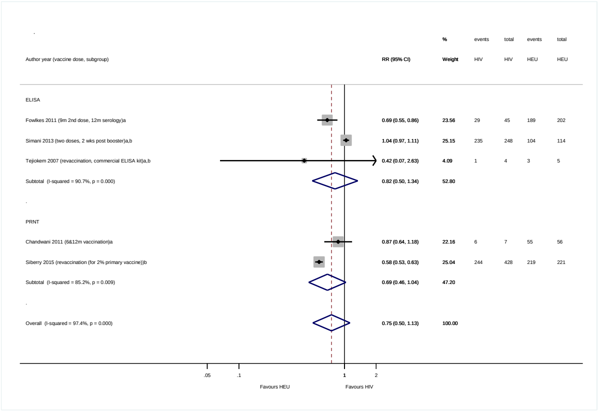

(C)

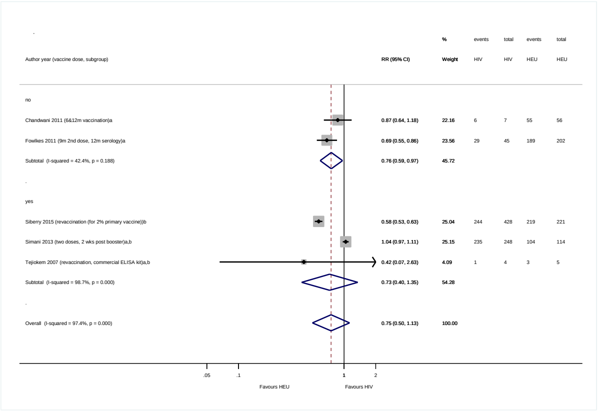

(D)

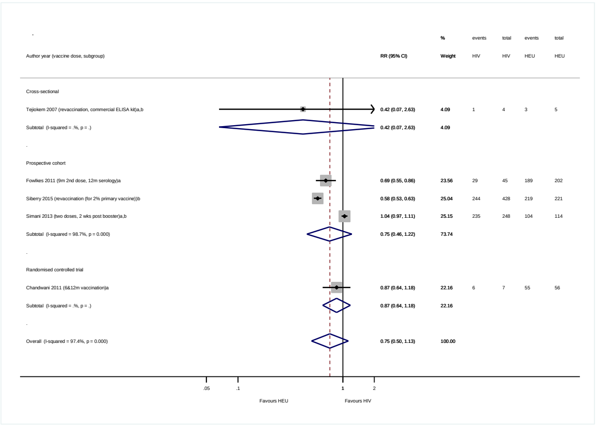

(E)

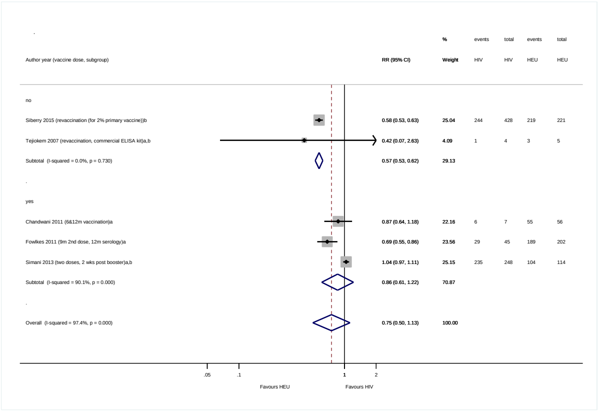

Supplementary data 8.5: Meta-analyses comparing HIV-exposed uninfected and HIV-unexposed children after primary measles vaccination stratified by (A) serological outcome, (B) serological assay to measure antibody response, (C) study design, and (D) time interval between vaccination and measurement of serology within 6 months

(A)

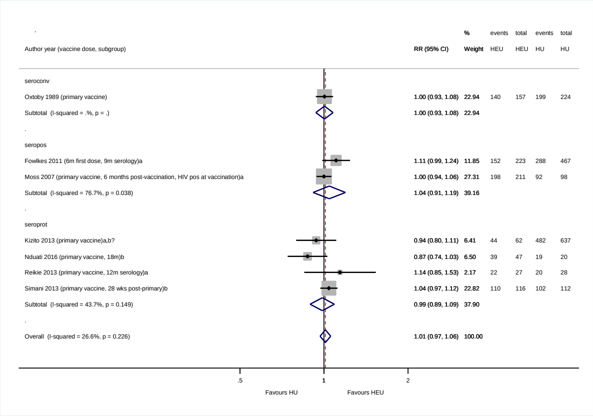

(B)

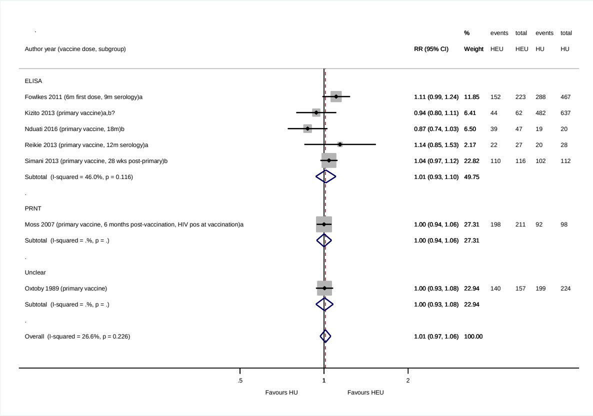

(C)

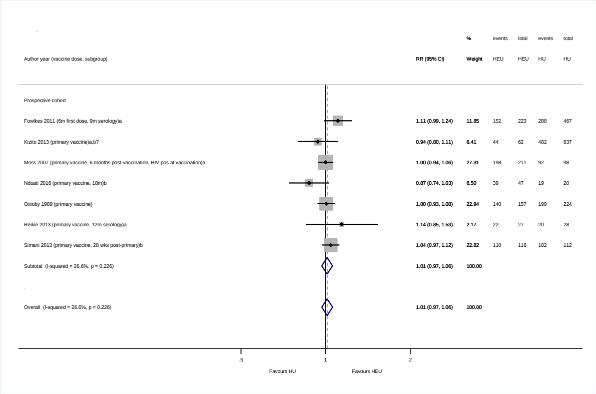

(D)

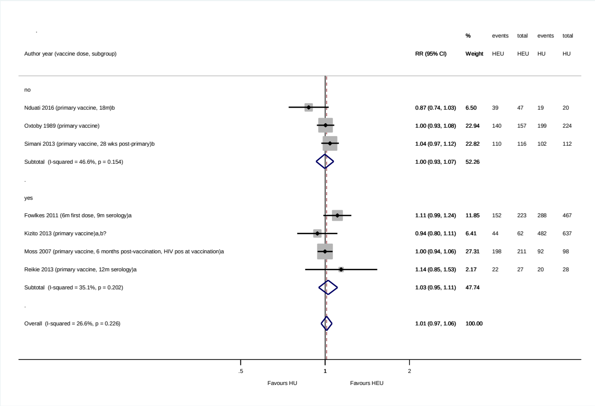

Supplementary data 9: Safety – adverse events

Twelve reports explicitly mentioned the number of adverse events (AEs) in HIV-infected children after measles vaccination. Overall, from 3,210 children evaluated, 1,096 experienced adverse events (34%); AEs were experienced by 192 of 575 (33%; median 40%; IQR 7%-54%) HIV-infected children, 284 of 1128 (25%; median 31%; IQR 11%-35%) HEU children and by significantly more HIV-unexposed children 620 of 1506 (41%; median 39%; IQR 36%-45%) (HIV-uninfected children with unknown exposure status counted as HEU[7]) ($p<0·001$). However, three studies reported that HIV-infected children were more likely to experience fever, cough and diarrhoea within 5-15 days,[7] 21 days,[11,12] or 1 month[21] post-vaccination compared with HIV-uninfected children.

Supplementary data 10: Risk of bias assessment for included studies by study design
Prospective cohort studies(n=32, retrospective cohort studies(n=3), and randomised controlled trial(n=1)

	Selection of study population	Incomplete outcome data (attrition bias)	Origin of data	Clarity of outcome definition	Consideration of confounders
Abzug 2012	?	+	+	+	+
Aurpibul 2007	−	+	+	+	?
Bekker 2006	?	?	+	+	?
Cagigi 2014	−	?	+	+	+
Chandwani 2011 (& Chandwani 1998)	+	+	+	+	+
Cutts 1993	+	−	+	+	?
Dunn 1998	?	?	−	?	−
Farquhar 2009	+	+	+	+	?
Fernandez-Ibieta 2007	?	−	?	?	−
Fowlkes 2011 (& Helfand 2008)	+	−	+	+	?
Fowlkes 2016	+	−	+	+	?
Hilgartner 2001	−	?	?	+	?
Jain 2017	+	?	+	+	+
Kizito 2013	+	?	+	+	?
Lepage 1992	+	−	+	+	?
Marczyžska 2001	?	?	+	+	+
McLaughlin 1988	−	?	−	?	?
Melvin 2003	−	?	+	+	?
Moss 2007	+	+	+	+	?
Nair 2009	−	?	+	−	?
Nduati 2016 (& Nduati 2012)	+	+	+	+	?
Newman 2017 (& Newman 2015)	+	+	+	+	?
Oldakowska 2001	?	?	+	+	?
Oldakowska 2008	?	?	?	?	+
Omenda 2015	−	?	+	?	−
Oxtoby 1989	?	?	?	?	−
Palumbo 1992 (& Hoyt 1992)	+	?	+	+	?
Rainwater-Lovett 2013	+	+	+	+	?
Reikie 2013	+	+	+	+	?
Seth 2016	+	?	+	+	?
Siberry 2015	+	?	+	+	+
Simani 2013	+	−	+	+	?
Succi 2018	+	+	+	+	?
Sudfeld 2013	+	?	+	+	?
Takano 2003	?	?	+	+	?
Thaithumyanon 2000	?	+	+	+	?

Cross-sectional studies (n=17), prospective cohort/cross-sectional studies(n=3), retrospective cohort/cross-sectional studies(n=10)

	Selection of study population	Incomplete outcome data (attrition bias)	Origin of data	Clarity of outcome definition	Consideration of confounders
al-Attar 1995	+	?	+	+	+
Arpadi 1996 (& Arpadi 1992)	+	?	+	+	+
Aurpibul 2006	?	?	+	+	+
Berkelhamer 2001	+	?	+	+	?
Brena 1993	−	?	+	+	?
Cardemil 2016	+	?	+	+	?
Echeverria 1996	+	?	+	+	?
Fitter 2013	+	?	+	+	?
Frenkel 1994 (& Frenkel 1992)	?	?	+	−	+
Lindgren-Alves 2001	+	?	+	+	?
Lowther 2009	+	+	+	+	?
Lyamuya 1999	+	?	+	+	?
Molyneaux 1993	+	+	+	+	−
Morris 2015	+	+	+	+	?
Myers 2009	+	+	+	+	?
Ndikuyeze 1987	?	?	+	−	?
Newman 2014	+	?	+	+	?
Oshitani 1996	+	?	+	−	?
Pensieroso 2009	+	?	+	+	?
Polonsky 2015 (& Polonsky 2015)	+	?	+	+	?
Rosso 2011	?	?	?	?	−
Rowson 2015	−	?	+	?	?
Rudy 1994	+	?	+	?	?
Ruel 2008 (& Ruel 2007)	+	?	+	+	?
Singh 2013	?	?	−	?	?
Sticchi 2015	+	?	?	?	?
Sutcliffe 2016	+	?	+	+	?
Tejiokem 2007	+	?	+	+	+
Waibale 1999	+	?	+	+	−
Walter 1994	−	?	+	+	?

Other: case reports(n=2), retrospective audit(n=1), unclear(n=2)

	Selection of study population	Incomplete outcome data (attrition bias)	Origin of data	Clarity of outcome definition	Consideration of confounders
Brunell 1995	−	?	+	+	?
Dhesi 2012	?		−	?	−
Embree 1989	?	?	?	?	−
Goon 2001	−		+	+	
Ramon-Garcia 1995	−		+	+	

Supplementary data 11: GRADE quality of evidence for immunogenicity of measles vaccination in HIV-infected and HIV-unexposed children

Certainty assessment							№ of patients		Effect		Certainty	Importance
**№ of studies**	**Study design**	**Risk of bias**	**Inconsistency**	**Indirectness**	**Imprecision**	**Other considerations**	**HIV-infected children**	**HIV-unexposed children**	**Relative (95% CI)**	**Absolute (95% CI)**		
Seroresponses comparing HIV-infected and HIV-unexposed children after one dose of measles vaccine												
9	observational studies	not serious	serious [a]	not serious	not serious	publication bias strongly suspected [b]	425/617 (68.9%)	1860/2284 (81.4%)	**RR 0.74** (0.61 to 0.90)	**212 fewer per 1,000** (from 81 fewer to 318 fewer)	⊕◯◯◯ VERY LOW	IMPORTANT
Seroresponses comparing HIV-infected and HIV-unexposed children after two doses of measles vaccine												
5	observational studies	not serious	serious [c]	not serious	not serious	none	306/346 (88.4%)	649/689 (94.2%)	**RR 0.84** (0.68 to 1.04)	**151 fewer per 1,000** (from 38 more to 301 fewer)	⊕◯◯◯ VERY LOW	IMPORTANT

Abbreviations: CI, Confidence interval; RR, Risk ratio;
[a] Considerable heterogeneity (I^2=89.5%);
[b] Funnel plot asymmetry;
[c] Considerable heterogeneity (I^2=89.6%).

Supplementary data 12: GRADE quality of evidence for immunogenicity of measles vaccination in HIV-infected and HIV-exposed uninfected children

Certainty assessment							№ of patients		Effect		Certainty	Importance
**№ of studies**	**Study design**	**Risk of bias**	**Inconsistency**	**Indirectness**	**Imprecision**	**Other considerations**	**HIV-infected children**	**HIV-exposed uninfected children**	**Relative (95% CI)**	**Absolute (95% CI)**		
Seroresponses comparing HIV-infected and HIV-exposed uninfected children after one dose of measles vaccine												
19	observational studies	not serious [a]	serious [b]	not serious	not serious	publication bias strongly suspected [c]	553/790 (70.0%)	1194/1515 (78.8%)	**RR 0.76** (0.67 to 0.86)	**189 fewer per 1,000** (from 110 fewer to 260 fewer)	⨁◯◯◯ VERY LOW	IMPORTANT
1	randomised trials	not serious	not serious	not serious	not serious	Only one study available; small sample size	14/14 (100.0%)	71/83 (85.5%)	**RR 1.17** (0.94 to 1.46)	**145 more per 1,000** (from 51 fewer to 393 more)	⨁⨁⨁◯ MODERATE	IMPORTANT
Seroresponses comparing HIV-infected and HIV-exposed uninfected children after two doses of measles vaccine												
4	observational studies	not serious	serious [d]	not serious	not serious	none	509/725 (70.2%)	515/542 (95.0%)	**RR 0.72** (0.45 to 1.17)	**266 fewer per 1,000** (from 162 more to 523 fewer)	⨁◯◯◯ VERY LOW	IMPORTANT
1	randomised trials	not serious	not serious	not serious	not serious	Only one study available; small sample size	6/7 (85.7%)	55/56 (98.2%)	**RR 0.87** (0.64 to 1.18)	**128 fewer per 1,000** (from 177 more to 354 fewer)	⨁⨁⨁◯ MODERATE	IMPORTANT

Abbreviations: CI, Confidence interval; RR, Risk ratio;
[a] Unclear Risk of Bias (Not Downgraded);
[b] Considerable heterogeneity (I^2=75.5%);
[c] Funnel plot asymmetry;
[d] Considerable heterogeneity (I^2=98.0%).

Supplementary data 13: GRADE quality of evidence for immunogenicity of measles vaccination in HIV-exposed uninfected and HIV-unexposed children

Certainty assessment							№ of patients		Effect		Certainty	Importance
**№ of studies**	**Study design**	**Risk of bias**	**Inconsistency**	**Indirectness**	**Imprecision**	**Other considerations**	**HIV-exposed uninfected children**	**HIV-unexposed children**	**Relative (95% CI)**	**Absolute (95% CI)**		
Seroresponses comparing HIV-exposed uninfected and HIV-unexposed children after one dose of measles vaccine												
7	observational studies	not serious	not serious	not serious	not serious	none	705/843 (83.6%)	1202/1586 (75.8%)	**RR 1.03** (0.98 to 1.07)	**23 more per 1,000** (from 15 fewer to 53 more)	⨁⨁◯◯ LOW	IMPORTANT
Seroresponses comparing HIV-exposed uninfected and HIV-unexposed children after two doses of measles vaccine												
3	observational studies	not serious	serious [a]	not serious	not serious	Very few studies available	312/343 (91.0%)	509/559 (91.1%)	**RR 0.99** (0.91 to 1.09)	**9 fewer per 1,000** (from 82 fewer to 82 more)	⨁◯◯◯ VERY LOW	IMPORTANT

Abbreviations: CI, Confidence interval; RR, Risk ratio;
[a] Moderate heterogeneity (I^2=67.7%).

Supplementary data 14: GRADE quality of evidence for safety of measles vaccination in HIV-infected and HIV-exposed children

Certainty assessment							№ of patients		Effect		Certainty	Importance
**№ of studies**	**Study design**	**Risk of bias**	**Inconsistency**	**Indirectness**	**Imprecision**	**Other considerations**	**[intervention]**	**[comparison]**	**Relative (95% CI)**	**Absolute (95% CI)**		
Safety of measles vaccination in HIV-infected children												
26	observational studies	serious [a]	not serious	not serious	not serious	publication bias strongly suspected [b]	#	#	#	#	⨁◯◯◯ VERY LOW	IMPORTANT
Safety of measles vaccination in HIV-exposed uninfected children												
11	observational studies	serious [a]	not serious	not serious	not serious	publication bias strongly suspected [c]	#	#	#	#	⨁◯◯◯ VERY LOW	IMPORTANT

Abbreviations: CI, Confidence interval;
[a] Absence of direct comparisons between vaccinated and unvaccinated HIV-infected children and poor quality of reporting;
[b] Incoherent safety reporting; HIV-infected children may experience more SAEs due to their underlying illness, unrelated to vaccine administration;
[c] Incoherent safety reporting;
No summary estimates per intervention or control group are applicable.

Supplementary data 15: (A) Funnel plot HIV-infected vs HEU children after primary measles vaccination; (B) Contour-enhanced funnel plot HIV-infected vs HEU children after primary measles vaccination.

(A)

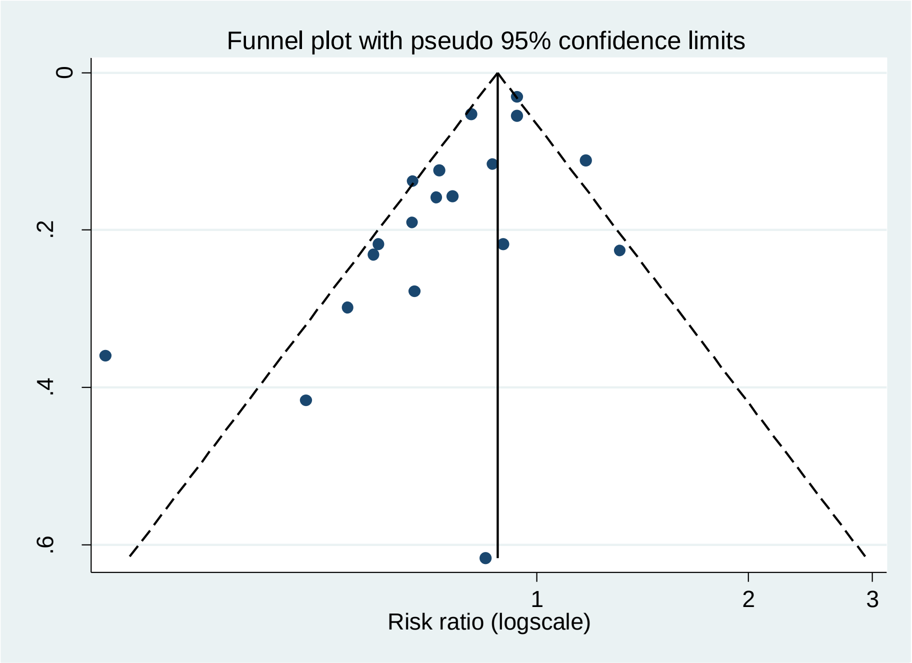

(B)

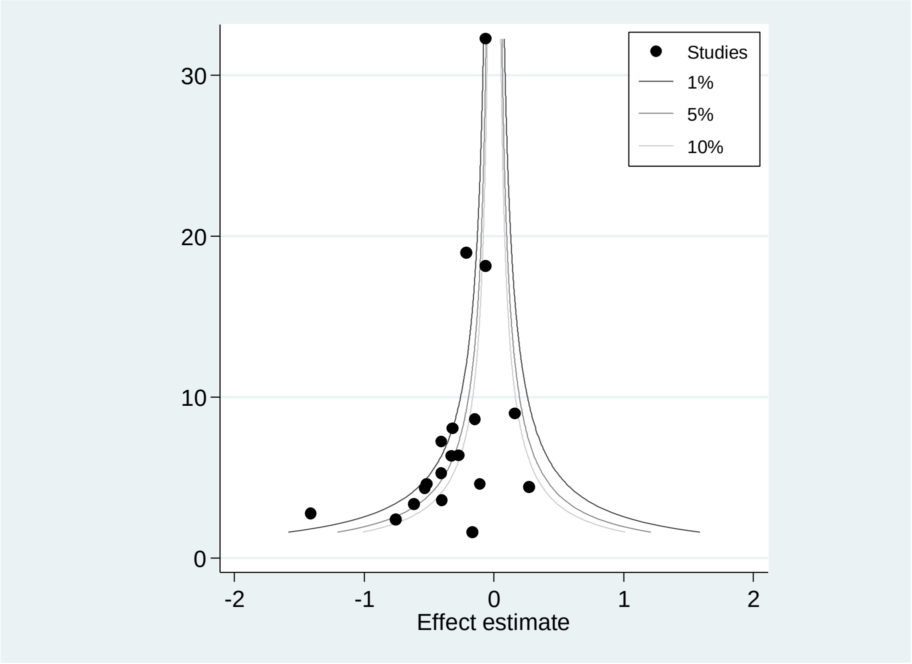

References supplementary data

References

1. Abzug MJ, Qin M, Levin MJ, et al. Immunogenicity, immunologic memory, and safety following measles revaccination in HIV-infected children receiving highly active antiretroviral therapy. *J Infect Dis* 2012; **206**(4): 512-22.

2. Aurpibul L, Puthanakit T, Sirisanthana T, Sirisanthana V. Response to measles, mumps, and rubella revaccination in HIV-infected children with immune recovery after highly active antiretroviral therapy. 2007; **45**: 637-42.

3. Bekker V, Scherpbier H, Pajkrt D, Jurriaans S, Zaaijer H, Kuijpers TW. Persistent humoral immune defect in highly active antiretroviral therapy-treated children with HIV-1 infection: loss of specific antibodies against attenuated vaccine strains and natural viral infection. *Pediatrics* 2006; **118**(2): e315-22.

4. Cagigi A, Rinaldi S, Cotugno N, et al. Early highly active antiretroviral therapy enhances B-cell longevity: A 5 year follow up. *Pediatr Infect Dis J* 2014; **33**(5): e126-31.

5. Chandwani S, Beeler J, Li H, et al. Safety and immunogenicity of early measles vaccination in children born to HIV-infected mothers in the United States: results of Pediatric AIDS Clinical Trials Group (PACTG) protocol 225. *J Infect Dis* 2011; **204 Suppl 1**: S179-89.

6. Chandwani S, Beeler J, Yang I, Moye J, Smith B, Nevin T, Krasinski K. Immunogenicity and safety of early measles vaccination in children born to HIV-infected mothers: results of pediatric AIDS clinical trials group (PACTG) protocol 225. *Intersci Conf Antimicrob Agents Chemother* 1998; **Sep 24-27**(38:366).

7. Cutts FT, Mandala K, St Louis M, et al. Immunogenicity of high-titer Edmonston-Zagreb measles vaccine in human immunodeficiency virus-infected children in Kinshasa, Zaire. *J Infect Dis* 1993; **167**(6): 1418-21.

8. Dunn DT, Newell ML, Peckham CS, Vanden Eijden S. Routine vaccination and vaccine-preventable infections in children born to human immunodeficiency virus-infected mothers. European Collaborative Study. *Acta Paediatr* 1998; **87**(4): 458-9.

9. Farquhar C, Wamalwa D, Selig S, et al. Immune responses to measles and tetanus vaccines among Kenyan human immunodeficiency virus type 1 (HIV-1)-infected children pre- and post-highly active antiretroviral therapy and revaccination. *Pediatr Infect Dis J* 2009; **28**(4): 295-9.

10. Fernandez-Ibieta M, Ramos-Amador J, Aunon-Martin I. HIV-infected children vaccination coverage and safety in a Western European cohort: A retrospective study. *Int J STD AIDS* 2007; **18**(5): 351-3.

11. Fowlkes A, Witte D, Beeler J, et al. Persistence of vaccine-induced measles antibody beyond age 12 months: a comparison of response to one and two doses of Edmonston-Zagreb measles vaccine among HIV-infected and uninfected children in Malawi. *J Infect Dis* 2011; **204 Suppl 1**: S149-57. doi:10.1093/infdis/jir135 [doi].

12. Helfand RF, Witte D, Fowlkes A, et al. Evaluation of the immune response to a 2-dose measles vaccination schedule administered at 6 and 9 months of age to HIV-infected and HIV-uninfected children in Malawi. *J Infect Dis* 2008; **198**(10): 1457-65. doi:10.1086/592756 [doi].

13. Fowlkes AL, Witte D, Beeler J, et al. Supplemental measles vaccine antibody response among HIV-infected and -uninfected children in Malawi after 1- and 2-dose primary measles vaccination schedules. *Vaccine* 2016; **34**(12): 1459-64.

14. Hilgartner MW, Maeder MA, Mahoney EM, Donfield SM, Evatt BL, Hoots WK. Response to measles, mumps, and rubella revaccination among HIV-positive and HIV-negative children and adolescents with hemophilia. *Am J Hematol* 2001; **66**(2): 92-8.

15. Jain S, Seth A, Khare S, Chandra J. Seroprevalence of transplacentally acquired measles antibodies in HIV-exposed versus HIV-unexposed infants at six months of age. *Indian J Med Res* 2017; **145**(4): 536-42. doi:10.4103/ijmr.IJMR_44_16 [doi].

16. Kizito D, Tweyongyere R, Namatovu A, et al. Factors affecting the infant antibody response to measles immunisation in Entebbe-Uganda. *BMC Public Health* 2013; **13**: 619,2458-13-619. doi:10.1186/1471-2458-13-619 [doi].

17. Lepage P, Dabis F, Msellati P, et al. Safety and immunogenicity of high-dose Edmonston-Zagreb measles vaccine in children with HIV-1 infection. A cohort study in Kigali, Rwanda. *Am J Dis Child* 1992; **146**(5): 550-5.

18. Marczynska M, Oldakowska A, Szczepanska-Putz M. Measles antibody in vaccinated HIV-infected children and effects of measles revaccination. *Cent -Eur J Immunol* 2001; **26**(2): 69-71.

19. McLaughlin M, Thomas P, Onorato I, et al. Live virus vaccines in human immunodeficiency virus-infected children: a retrospective survey. *Pediatrics* 1988; **82**(2): 229-33.

20. Melvin AJ, Mohan KM. Response to immunization with measles, tetanus, and Haemophilus influenzae type b vaccines in children who have human immunodeficiency virus type 1 infection and are treated with highly active antiretroviral therapy. *Pediatrics* 2003; **111**(6): e641-4.

21. Moss WJ, Scott S, Mugala N, et al. Immunogenicity of standard-titer measles vaccine in HIV-1-infected and uninfected zambian children: an observational study. *J Infect Dis* 2007; **196**(3): 347-55.

22. Nair N, Moss WJ, Scott S, et al. HIV-1 infection in Zambian children impairs the development and avidity maturation of measles virus-specific immunoglobulin G after vaccination and infection. *J Infect Dis* 2009; **200**(7): 1031-8.

23. Nduati EW, Nkumama IN, Gambo FK, et al. HIV-Exposed Uninfected Infants Show Robust Memory B-Cell Responses in Spite of a Delayed Accumulation of Memory B Cells: an Observational Study in the First 2 Years of Life. *Clin Vaccine Immunol* 2016; **23**(7): 576-85. doi:10.1128/CVI.00149-16 [doi].

24. Nduati EW, Marsh K, Urban B. Exposed but uninfected: Does HIV exposure alter immune responses in infants? *Am J Trop Med Hyg* 2012; **87**(5): 344.

25. Newman LP, Njoroge A, Magaret A, et al. Sustained Responses to Measles Revaccination at 24 Months in HIV-Infected Children on Antiretroviral Therapy in Kenya. *Pediatr Infect Dis J* 2017; . doi:10.1097/INF.0000000000001572 [doi].

26. Newman L, Njoroge A, Chohan B, et al. Sustained responses to measles revaccination in HIV-infected children on ART in Kenya. *Top Antiviral Med* 2015; **23**: 435.

27. Oldakowska A, Marczynska M, Szczepanska-Putz M. Effects of measles vaccination in HIV infected children. *Przegl Epidemiol* 2001; **55**(4): 523-7.

28. Oldakowska A, Marczynska M. Measles vaccination in HIV infected children]. *Med Wieku Rozwoj* 2008; **12**(2): 675-80.

29. Omenda MM. HIV-1 exposed uninfected and unexposed infants have similar antibody responses to childhood vaccines, but the responses against hepatitis B vaccine decay by twenty one months of age. *Am J Trop Med Hyg* 2015; **93**(4): 146.

30. Oxtoby MJ, Ryder R, Mvula M, Nsa W, Baende E, Onorato I. Patterns of Immunity to Measles Among African Children Infected with Human Immunodeficiency Virus. *In: Program and abstracts of Epidemic Intelligence Service Conference* 1989; **Atlanta: Centers for Disease Control**.

31. Palumbo P, Hoyt L, Demasio K, Oleske J, Connor E. Population-based study of measles and measles immunization in human immunodeficiency virus-infected children. *Pediatr Infect Dis J* 1992; **11**(12): 1008-14.

32. Hoyt L. Measles vaccine response and clinical measles in HIV-infected children. *Int Conf AIDS* 1992; **8**(1): Tu29.

33. Rainwater-Lovett K, Nkamba HC, Mubiana-Mbewe M, Bolton-Moore C, Moss WJ. Changes in measles serostatus among HIV-infected Zambian children initiating antiretroviral therapy before and after the 2010 measles outbreak and supplemental immunization activities. *J Infect Dis* 2013; **208**(11): 1747-55. doi:10.1093/infdis/jit404 [doi].

34. Reikie BA, Naidoo S, Ruck CE, et al. Antibody responses to vaccination among South African HIV-exposed and unexposed uninfected infants during the first 2 years of life. *Clin Vaccine Immunol* 2013; **20**(1): 33-8. doi:10.1128/CVI.00557-12 [doi].

35. Seth A, Deepa S, Dutta R, Chandra J. Evaluation of Immune Response to Measles Component of MMR Vaccine in Children with HIV Infection Receiving Antiretroviral Therapy. *Pediatr Infect Dis J* 2016; **35**(1): e8-11. doi:10.1097/INF.0000000000000934 [doi].

36. Siberry GK, Patel K, Bellini WJ, et al. Immunity to Measles, Mumps, and Rubella in US Children With Perinatal HIV Infection or Perinatal HIV Exposure Without Infection. *Clin Infect Dis* 2015; **61**(6): 988-95.

37. Simani OE, Adrian PV, Violari A, et al. Effect of in-utero HIV exposure and antiretroviral treatment strategies on measles susceptibility and immunogenicity of measles vaccine. *AIDS* 2013; **27**(10): 1583-91. doi:10.1097/QAD.0b013e32835fae26 [doi].

38. Succi RCM, Krauss MR, Harris DR, et al. Immunity After Childhood Vaccinations in Perinatally HIV-exposed Children With and Without HIV Infection in Latin America. *Pediatr Infect Dis J* 2018; **37**(4): 304-9. doi:10.1097/INF.0000000000001831 [doi].

39. Sudfeld CR, Duggan C, Histed A, et al. Effect of multivitamin supplementation on measles vaccine response among HIV-exposed uninfected Tanzanian infants. *Clin Vaccine Immunol* 2013; **20**(8): 1123-32. doi:10.1128/CVI.00183-13 [doi].

40. Takano D, Russo P, Rufino A, Succi R, Weckx L, De Moraes-Pinto MI. Measles and Rubella Antibodies in Fully Immunized HIV-1 Infected Children: Response to an Extra MMR Dose under HAART [Paper 780]. *10th Conference on Retroviruses and Opportunistic Infections* 2003; **Bston, MA**.

41. Thaithumyanon P, Punnahitananda S, Thisyakorn U, Praisuwanna P, Ruxrungtham K. Immune responses to measles immunization and the impacts on HIV-infected children. *Southeast Asian J Trop Med Public Health* 2000; **31**(4): 658-62.

42. al-Attar I, Reisman J, Muehlmann M, McIntosh K. Decline of measles antibody titers after immunization in human immunodeficiency virus-infected children. *Pediatr Infect Dis J* 1995; **14**(2): 149-51.

43. Arpadi SM, Markowitz LE, Baughman AL, et al. Measles antibody in vaccinated human immunodeficiency virus type 1-infected children. *Pediatrics* 1996; **97**(5): 653-7.

44. Arpadi SM. Measles antibody in vaccinated HIV-infected children. *Int Conf AIDS* 1992; **8**(2): B203.

45. Aurpibul L, Puthanakit T, Siriaksorn S, Sirisanthana T, Sirisanthana V. Prevalence of protective antibody against measles in HIV-infected children with immune recovery after highly active antiretroviral therapy. *HIV Med* 2006; **7**(7): 467-70.

46. Berkelhamer S, Borock E, Elsen C, Englund J, Johnson D. Effect of highly active antiretroviral therapy on the serological response to additional measles vaccinations in human immunodeficiency virus-infected children. *Clin Infect Dis* 2001; **32**(7): 1090-4. doi:CID000249 [pii].

47. Brena AE, Cooper ER, Cabral HJ, Pelton SI. Antibody response to measles and rubella vaccine by children with HIV infection. *J Acquir Immune Defic Syndr* 1993; **6**(10): 1125-9.

48. Cardemil CV, Jonas A, Beukes A, et al. Measles immunity among pregnant women aged 15-44 years in Namibia, 2008 and 2010. *Int J Infect Dis* 2016; **49**: 189-95.

49. Echeverria Lecuona J, Aldamiz-Echevarria Azuara L, Cilla Eguiluz G, Perez Trallero E. Responses to triple viral and tetanus vaccination in HIV-infected children. *An Esp Pediatr* 1996; **44**(4): 317-20.

50. Fitter DL, Anselme R, Paluku G, et al. Seroprevalence of measles and rubella antibodies in pregnant women Haiti, 2012. *Vaccine* 2013; **32**(1): 69-73.

51. Frenkel LM, Nielsen K, Garakian A, Cherry JD. A search for persistent measles, mumps, and rubella vaccine virus in children with human immunodeficiency virus type 1 infection. *Arch Pediatr Adolesc Med* 1994; **148**(1): 57-60.

52. Frenkel LM. Evaluation of the persistence of MMR vaccine viruses in HIV-infected P2 children. *Int Conf AIDS* 1992; **8**(2): B197.

53. Lindgren-Alves CR, Freire LM, Oliveira RC, et al. Search of antimeasles antibodies in HIV-infected children after basic immunization. *J Pediatr (Rio J)* 2001; **77**(6): 496-502.

54. Lowther SA, Curriero FC, Kalish BT, Shields TM, Monze M, Moss WJ. Population immunity to measles virus and the effect of HIV-1 infection after a mass measles vaccination campaign in Lusaka, Zambia: a cross-sectional survey. *Lancet* 2009; **373**(9668): 1025-32.

55. Lyamuya EF, Matee MIN, Aaby P, Scheutz F. Serum levels of measles IgG antibody activity in children under 5 years in Dar-es-Salaam, Tanzania. *Ann Trop Paediatr* 1999; **19**(2): 175-83.

56. Molyneaux PJ, Mok JYQ, Burns SM, Yap PL. Measles, mumps and rubella immunisation in children at risk of infection with human immunodeficiency virus. *J Infect* 1993; **27**(3): 251-3.

57. Morris LE, Posada R, Hickman CJ, et al. Susceptibility to measles among perinatally HIV-infected adolescents and young adults. *J Pediatric Infect Dis Soc* 2015; **4**(1): 63-6.

58. Myers C, Posfay-Barbe KM, Aebi C, et al. Determinants of vaccine immunity in the cohort of human immunodeficiency virus-infected children living in Switzerland. *Pediatr Infect Dis J* 2009; **28**(11): 996-1001. doi:10.1097/INF.0b013e3181a78348 [doi].

59. Ndikuyeze A, Taylor E, Farzadegan H, Polk BF. Measles immunization in children with human immunodeficiency virus infection. *Vaccine* 1987; **5**(3): 168.

60. Newman LP, Njoroge A, Ben-Youssef L, et al. Measles seropositivity in HIV-infected Kenyan children on antiretroviral therapy. *Pediatr Infect Dis J* 2014; **33**(8): 843-5.

61. Oshitani H, Suzuki I, Mpabalwani ME, Mizuta K, Numazaki Y. Measles case fatality by sex, vaccination status, and HIV-1 antibody in Zambian children 26]. *Lancet* 1996; **348**(9024): 415.

62. Pensieroso S, Cagigi A, Palma P, et al. Timing of HAART defines the integrity of memory B cells and the longevity of humoral responses in HIV-1 vertically-infected children. *Proc Natl Acad Sci U S A* 2009; **106**(19): 7939-44. doi:10.1073/pnas.0901702106 [doi].

63. Polonsky JA, Singh B, Masiku C, et al. Exploring HIV infection and susceptibility to measles among older children and adults in Malawi: a facility-based study. *Int J Infect Dis* 2015; **31**: 61-7. doi:10.1016/j.ijid.2014.12.010 [doi].

64. Polonsky JA, Juan-Giner A, Hurtado N, Masiku C, Kagoli M, Grais RF. Measles seroprevalence in Chiradzulu district, Malawi: Implications for evaluating vaccine coverage. *Vaccine* 2015; **33**(36): 4554-8.

65. Rosso R, De Hoffer L, Parisini A, et al. Responses to common vaccines in adolescents with HIV acquired perinatally. *Infection* 2011; **39**: S69-70.

66. Rowson K, Tan A, Donaghy S, Doerholt K, Heath P, Riordan A. Measles reimmunization may not be effective in protecting HIV-infected children. *Pediatr Infect Dis J* 2015; **34**(5): 552.

67. Rudy BJ, Rutstein RM, Pinto-Martin J. Responses to measles immunization in children infected with human immunodeficiency virus. *J Pediatr* 1994; **125**(1): 72-4. doi:S0022-3476(94)70125-3 [pii].

68. Ruel TD, Achan J, Gasasira AF, et al. HIV RNA suppression among HIV-infected Ugandan children with Measles. *J Acquired Immune Defic Syndr* 2008; **48**(2): 225-7.

69. Singh A, Rowson K, McMaster P, Tan A. Audit of MMR vaccine response in HIV-infected children in a region with a recent measles outbreak. *HIV Med* 2013; **14**: 33.

70. Sticchi L, Bruzzone B, Caligiuri P, et al. Seroprevalence and vaccination coverage of vaccine-preventable diseases in perinatally HIV-1-infected patients. *Hum Vaccin Immunother* 2015; **11**(1): 263-9.

71. Sutcliffe CG, Searle K, Matakala HK, et al. Measles and Rubella Seroprevalence Among HIV-Infected And Uninfected Zambian Youth. *Pediatr Infect Dis J* 2016; . doi:10.1097/INF.0000000000001422 [doi].

72. Tejiokem MC, Gouandjika I, Beniguel L, et al. HIV-infected children living in Central Africa have low persistence of antibodies to vaccines used in the Expanded Program on Immunization. *PLoS ONE* 2007; **2**(12): e1260.

73. Waibale P, Bowlin SJ, Mortimer EA,Jr, Whalen C. The effect of human immunodeficiency virus-1 infection and stunting on measles immunoglobulin-G levels in children vaccinated against measles in Uganda. *Int J Epidemiol* 1999; **28**(2): 341-6.

74. Walter EB, Katz SL, Bellini WJ. Measles immunity in HIV-infected children. *Pediatr AIDS HIV Infect* 1994; **5**(5): 300-4.

75. Brunell PA, Vimal V, Sandu M, Courville TM, Daar E, Israele V. Abnormalities of measles antibody response in human immunodeficiency virus type 1 (HIV-1) infection. *J Acquir Immune Defic Syndr Hum Retrovirol* 1995; **10**(5): 540-8.

76. Dhesi A, Bandi S, Blake K, Welch S, Thompson M. Immunisation of children with HIV. *Arch Dis Child* 2012; **97**: A32.

77. Embree JE. Safety and efficacy of immunizations with live vaccine. *Int Conf AIDS* 1989; **Jun 4-9**: 975 (abstract no. M.G.O.23).

78. Goon P, Cohen B, Jin L, Watkins R, Tudor-Williams G. MMR vaccine in HIV-infected children -- potential hazards? *Vaccine* 2001; **19**(28-29): 3816-9. doi:S0264-410X(01)00122-0 [pii].

79. Ramon-Garcia G, De LC, Sadowinski-Pine S, Santos-Preciado J, Hernandez-Mote R, Valencia-Mayoral P. Measles in two patients with AIDS. *Bol Med Hosp Infant Mex* 1995; **52**(2): 112-8.
